# Histone modification profiling in breast cancer cell lines highlights commonalities and differences among subtypes

**DOI:** 10.1186/s12864-018-4533-0

**Published:** 2018-02-20

**Authors:** Yuanxin Xi, Jiejun Shi, Wenqian Li, Kaori Tanaka, Kendra L. Allton, Dana Richardson, Jing Li, Hector L. Franco, Anusha Nagari, Venkat S. Malladi, Luis Della Coletta, Melissa S. Simper, Khandan Keyomarsi, Jianjun Shen, Mark T. Bedford, Xiaobing Shi, Michelle C. Barton, W. Lee Kraus, Wei Li, Sharon Y. R. Dent

**Affiliations:** 1 Department of Molecular and Cellular Biology and Division of Biostatistics, Dan L. Duncan Cancer Center, Baylor College of Medicine, Houston, Texas 77030 USA; 2 The Department of Epigenetics and Molecular Carcinogenesis, University of Texas Graduate School of Biomedical Sciences at Houston and The Center for Cancer Epigenetics, University of Texas M.D. Anderson Cancer Center, Houston, Texas 77030 USA; 3 The Department of Experimental Radiation Oncology, University of Texas MD Anderson Cancer Center, Houston, Texas 77030 USA; 4 Laboratory of Signaling and Gene Regulation, Cecil H. and Ida Green Center for Reproductive Biology Sciences and Division of Basic Reproductive Biology Research, Department of Obstetrics and Gynecology, University of Texas Southwestern Medical Center, Dallas, TX 75390 USA

**Keywords:** Breast cancer subtypes, Epigenetics, Histone modifications, Chromatin states

## Abstract

**Background:**

Epigenetic regulators are frequently mutated or aberrantly expressed in a variety of cancers, leading to altered transcription states that result in changes in cell identity, behavior, and response to therapy.

**Results:**

To define alterations in epigenetic landscapes in breast cancers, we profiled the distributions of 8 key histone modifications by ChIP-Seq, as well as primary (GRO-seq) and steady state (RNA-Seq) transcriptomes, across 13 distinct cell lines that represent 5 molecular subtypes of breast cancer and immortalized human mammary epithelial cells.

**Discussion:**

Using combinatorial patterns of distinct histone modification signals, we defined subtype-specific chromatin signatures to nominate potential biomarkers. This approach identified AFAP1-AS1 as a triple negative breast cancer-specific gene associated with cell proliferation and epithelial-mesenchymal-transition. In addition, our chromatin mapping data in basal TNBC cell lines are consistent with gene expression patterns in TCGA that indicate decreased activity of the androgen receptor pathway but increased activity of the vitamin D biosynthesis pathway.

**Conclusions:**

Together, these datasets provide a comprehensive resource for histone modification profiles that define epigenetic landscapes and reveal key chromatin signatures in breast cancer cell line subtypes with potential to identify novel and actionable targets for treatment.

**Electronic supplementary material:**

The online version of this article (10.1186/s12864-018-4533-0) contains supplementary material, which is available to authorized users.

## Background

Chromatin remodeling factors and histone modifying enzymes are frequently mutated or aberrantly expressed in a wide variety of cancers, leading to altered transcription states that change cell identity, behavior, and response to therapy. In recent years, large efforts have systematically profiled epigenomes in various tissue types and diseases, including the ENCODE project [1–3] and the Roadmap Epigenomics consortium [4]. Despite these important resources, how epigenetic profiles contribute to subtype heterogeneity in specific types of cancer is far from clear.

**Fig. 1:**
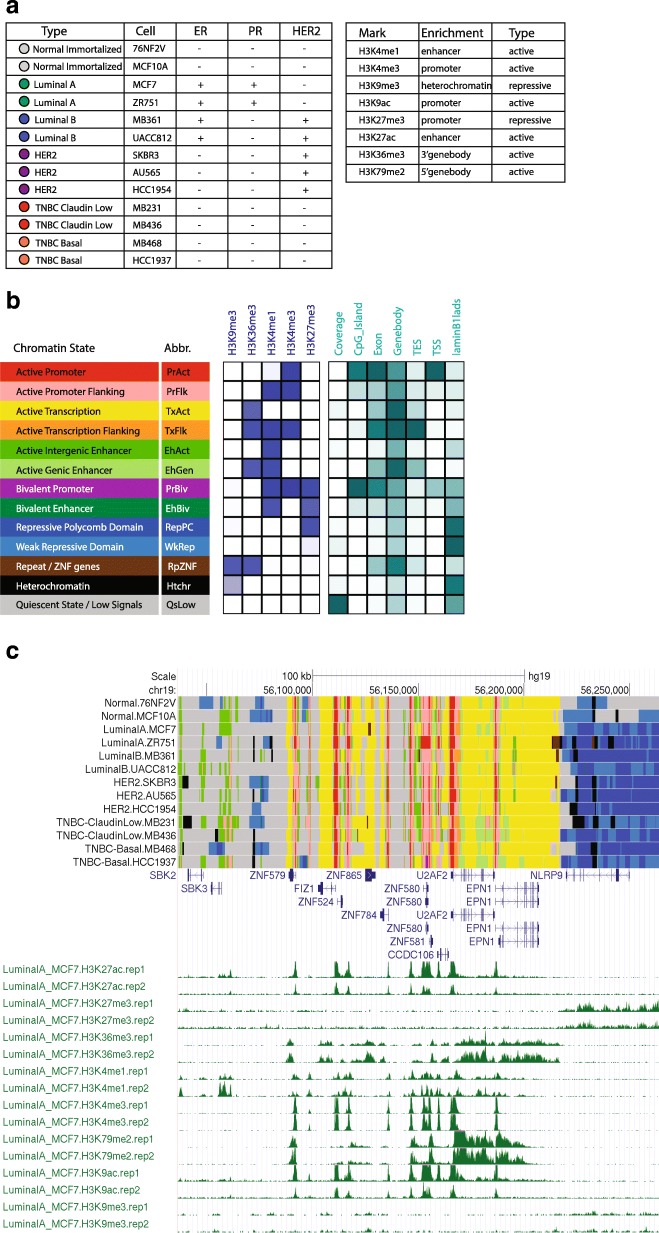
Cells and histone modifications collected in LONESTAR project. **a** Summary of cell lines and histone marks. **b** Thirteen chromatin states were defined using 5 key histone modifications, the left panel describes the chromatin state annotations and color scheme, the central panel describes the emission coefficients in ChromHMM model, the right panel describes the relative enrichment of coverage in whole genome and in different genomic regions. **c** Integrated view of whole genome chromatin state landscapes in breast cancer cells and corresponding individual histone modification profiles in MCF7 cells for a region on chromosome 19

Breast cancers are classified into at least 5 distinct molecular subtypes characterized by hormonal responses (e.g. estrogen and progesterone receptor status), growth factor expression (e.g. Her2/neu status), and specific gene expression profiles. The Lonestar Oncology Network for EpigeneticS Therapy And Research (LONESTAR) consortium was created to define epigenetic factors associated with molecular changes in specific subtypes of breast cancer. We reasoned that definition of subtype specific histone modification profiles, together with definition of primary and steady state transcriptional differences, may allow identification of new targets for development of precision therapies. Therefore, we profiled distributions of 8 key histone modifications by ChIP-Seq as well as primary (GRO-seq) and steady state (RNA-Seq) transcriptomes across 13 distinct cell lines that represent immortalized human mammary epithelial cells (hereafter referred to as normal immortalized or immortalized cells) and 5 molecular subtypes of breast cancer (Fig. 1a). These datasets provide a unique resource for breast cancer researchers, and reveal new insights into breast cancer biology. Highlights of our findings include:

- Definition of common and unique features of chromatin landscapes of all 5 breast cancer subtypes
- Demonstration that histone modifications associated with either enhancers or with active transcription states can discriminate between subtypes
- Identification of epigenetic landscapes that define genes and pathways specifically activated or repressed in TNBC

## Results

We systematically profiled histone modification patterns and gene expression programs in a set of well characterized cell lines that represent 5 major breast cancer subtypes, including two ER positive subtypes, Luminal-A and Luminal-B, the HER2 positive subtype, and two triple-negative subtypes, TNBC-Claudin Low, and TNBC-Basal, as well as two normal immortalized breast cell lines as controls (Fig. 1a; color codes indicated for each cell line will be used throughout the paper). For each cell line, we profiled 8 major histone modifications by ChIP-seq (Fig. 1a and Additional file 1: Figure S1), chosen for their known roles in transcriptional activation or repression. The whole dataset includes 234 ChIP-seq samples that contain a total of 12.8 billion sequencing reads (54.7 M reads per sample), with ~ 154 fold coverage of the human genome. All ChIP-seq experiments were performed in duplicate with good sequencing quality (Additional file 2: Table S1) and reproducibility between replicates (Additional file 3: Table S2). The ChIP-seq signals of these histone modifications show typical occupancy profiles at different genomic regions (e.g. promoter, enhancer, genebody, and heterochromatin; Additional file 1: Figure S1A-C), as well as whole genome scale distributions (Additional file 1: Figure S1D). In addition, to characterize the transcriptome profiles associated with the histone modifications, we also performed GRO-seq and RNA-seq for all of the cells lines in duplicate.

### Chromatin states

Previous studies by the NIH Roadmap Epigenetics consortium [4] defined chromatin states at characteristic loci using a Hidden-Markov-Model (HMM) based approach [5] by classifying the combinatorial patterns of 5 core histone modifications, including H3K4me3, H3K4me1, H3K27me3 and H3K9me3, and H3K36me3. Following this approach, we used the same 5 histone marks to define a 13-chromatin-state model from all 13 cell lines (Fig. 1b). This model was selected by examining the reproducibility of these 13 chromatin states on models trained on individual cell lines (Additional file 4: Figure S2A). According to the genomic distributions and histone modification enrichment patterns, these 13 chromatin states were annotated as active promoters (PrAct) and promoter flanking regions (PrFlk), active enhancers in intergenic regions (EhAct) and genic regions (EhGen), active transcription units (TxAct) and their flanking regions (TxFlk), strong (RepPC) and weak (WkREP) repressive polycomb domains, poised bivalent promoters (PrBiv) and bivalent enhancers (EhBiv), repeats/ZNF gene clusters (RpZNF), heterochromatin (Htchr), and quiescent/low signal regions (QsLow) (Fig. 1b). Together these 13 chromatin states represent the combinatorial histone modification patterns and define the whole genome chromatin state landscapes across the breast cancer cell lines. Combined with individual histone modification occupancy profiles, these chromatin state landscapes provide an integrated view of key epigenetic marks across all breast cancer cell lines and subtypes, as illustrated for a region on chromosome 19 (Fig. 1c).

In addition to the 5 core histone marks, we also profiled H3K27ac, H3K9ac and H3K79me2 distribution patterns across all cell lines. To validate the 13-chromatin-state model, an extended model was constructed using all 8 marks, in which the chromatin was classified into 15 states (Additional file 5: Figure S3). As expected, H3K9ac profiles coincide with those of H3K4me3, and H3K27ac is highly correlated with H3K4me1. Therefore, most chromatin states identified by the model built from analysis of 5 core histone modifications (Fig. 1b) were rebuilt in this expanded model (Additional file 5: Figure S3). Therefore, the 13-chromatin-state model was used in the following analyses.

Genes associated with different chromatin states show distinct transcriptional activities. For example, significantly higher expression levels were observed for loci enriched for chromatin states associated with active promoters (PrAct) and gene bodies (TxAct), as expected (Additional file 4: Figure S2B). The variability of each chromatin state was evaluated by their consistency at particular loci across all cell lines (Additional file 4: Figure S2C). We found that enhancer associated chromatin states, including intergenic enhancers (EhAct), gene body enhancers (EhGen) and bivalent enhancers (EhBiv), as well as repressive Polycomb domains (RepPC), display the most variability across different cell lines and subtypes, followed by chromatin states associated with active promoters (PrAct) and active transcription units (TxAct).

### Subtype specific signatures

**Fig. 2:**
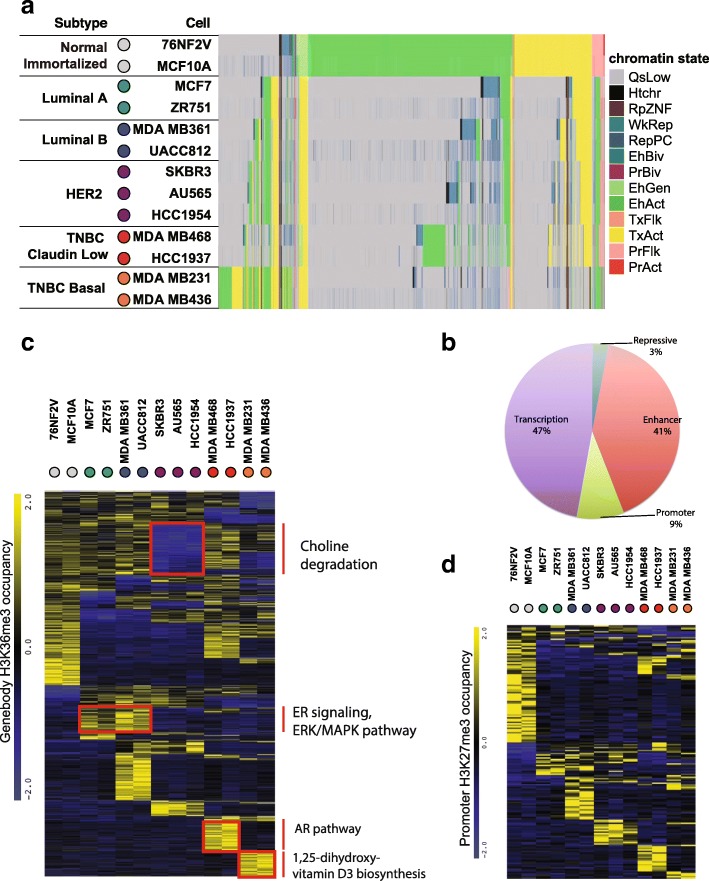
Chromatin states signatures for breast cancer subtypes. **a** Genome wide subtype specific patterns revealed by chromatin state clustering (chromatin state color scheme same as Fig. 1b). The Heatmap represents the top 1000 highly variable genomic regions clustered according to average linkage distance metric. **b** Pie chart of major chromatin states categories in subtype specific patterns across all cell lines. **c** Genebody H3K36me3 occupancy heatmap for genes associated with active transcription states. **d** Promoter H3K27me3 occupancy heatmap for genes associated with repressive states

We next determined whether specific chromatin state patterns were unique to one or several breast cancer subtypes. Through these analyses we identified common chromatin state patterns that distinguish individual subtypes from the other subtypes or from normal immortalized cells (Fig. 2a). The two normal immortalized cell lines, 76NF2V and MCF10A, displayed significantly distinct chromatin state distributions from all the breast cancer cell lines. The clustering structure further revealed hierarchical relationships among breast cancer subtypes. For example, a clear separation was observed between the hormone responsive subtypes (including Luminal A/B and HER2+) and triple negative subtypes (Claudin low and Basal). Luminal A and B subtypes were more closely related to each other than to normal immortal cells or to TNBC subtypes, while the epigenetic landscape in the Her2+ subtype cell lines was intermediate to that in the Luminal and TNBC subtypes. Within TNBC cells, the two basal TNBC cell lines, MB468 and HCC1937 clustered together, as did the two claudin low TNBC cell lines, MB231 and MB436. These subtype specific chromatin states were further confirmed by examining individual histone modification signals in different genomic regions, including promoters, enhancers and gene bodies (Additional file 6: Figure S4), and these data again showed clear subtype specificity in terms of characteristic histone modification patterns, with good reproducibility between replicates.

Active transcription-associated states, including both TxAct and TxFlk, made up the majority (47%) of chromatin states that were common between cell lines within the same subtype. Enhancer states (41%), including EhAct, EhGen and EhBiv, closely followed (Fig. 2b). The dynamic nature of enhancer associated chromatin states in breast cancer subtypes are further characterized by the enhancer RNA (eRNA) signals defined in the GRO-seq profiles (Additional file 7: Table S5), as will be presented in a separate paper from the LONESTAR consortium.

Since active transcription chromatin states, including both TxAct and TxFlk, represent genes with the highest expression levels (Additional file 4: Figure S2B), we first confirmed the strong prediction power of H3K36me3 on gene expression (Additional file 8: Figure S5), then examined H3K36me3 profiles (Fig. 2c) to more quantitatively characterize differential chromatin states associated with subtype specific patterns. We analyzed specific active and repressive transcription chromatin states for individual subtypes as well between subtype groups. As expected, estrogen signaling and ERK/MAPK pathways were identified as active in both ER-positive Luminal-A and Luminal-B subtypes (Fig. 2c). Several upstream regulators as defined by Ingenuity Pathway Analysis, including VPS36, HSP90B1, BCAR1, ASH2L and PDC6DIP, were also identified as active specifically in these cells. In contrast, several micro RNAs, including mir-103, mir-141-3p, mir130 and mir-22, and HAVCR1 were identified as negative upstream regulators (Additional file 9: Table S4, Additional file 10: Table S7, Additional file 11: Table S8, Additional file 12: Table S9) in ER positive cells. These analyses also identified some distinct regulators in Luminal A and Luminal B subtypes. For example, the arginine methyltransferase PRMT6 and the Hippo signaling associated genes ITCH and TEAD were identified as significant regulators of Luminal-A specific genes enriched in H3K36me3, while for Luminal-B cells, HOXB3, ESRP1 and WASL were identified as the most significant common upstream regulators for genes enriched in H3K4me3 (Additional file 12: Table S9).

For the HER2 positive subtype, a set of genes involved with choline degradation was identified among the genes depleted with the H3K36me3 signature. Choline has been identified as a breast cancer biomarker, and a recent study suggested distinct choline regulation in xenograft models of different breast cancer subtypes [6, 7]. Our results confirm this subtype specificity and further suggest specific dysregulation of choline metabolism in HER2 positive breast cancers. In addition, we validated that Choline dehydrogenase (CHDH) has significant lower expression in HER2 positive breast cancers in TCGA patient samples (Additional file 13: Figure S6). On the other hand, several WD-40 repeat containing genes, including CORO2A, TBL1X, TBL1XR1 were identified as common regulators of genes with an active signature in HER2 positive cells. As TBL1X and TBL1XR1 are components of the NCoR repressor complex, activity of these genes suggest that NCoR functions may be disrupted in these cells.

The basal-like and claudin-low TNBC subtypes display distinct H3K36me3 patterns (Fig. 2c). Interestingly, androgen receptor (AR) pathway genes were identified as active specifically in claudin-low cells. Consistent with this finding, we observed several AR pathway regulators are expressed at significantly lower levels in TCGA basal subtype patients compared with other subtype breast cancer patients, including AR, CREB3L1, CREB3L4, EGF, PDGFB, PDGFRB, PIK3R1, PTEN, RB1, ZEB1 (Additional file 13: Figure S6). Many of these genes are also well known tumor suppressor genes. Genes involved in 1,25-dihydroxyvitamin D3 biosynthesis were identified as active specifically in basal cells, consistent with up regulation of vitamin D 24-Hydrolase CYP24A1 in TCGA basal subtype patients (Additional file 13: Figure S6), and the top regulators identified included factors involved in cell-cycle regulation and signaling pathways of apoptosis and immune responses, including THAP1, ZBTB49, CASP3 and PAWR (Additional file 12: Table S9).

### Repressive chromatin states

Next we examined subtype specific repressive chromatin states, including polycomb domains (RepPC and WkRep), repeats/ZNF gene clusters (RpZNF) and heterochromatin (Htchr) (Figs. 1b and 2d). These repressive states are characterized by enriched H3K27me3 or H3K9me3 modification in the chromatin state models. H3K27me3 signals also define both bivalent domains in promoters (PrBiv) and enhancers (EhBiv), which are known for their poised activities during cell development as well as cancer progression. Genes associated with these repressive chromatin states show overall low expression levels in breast cancer cells (Additional file 4: Figure S2C). Similar to our analyses of active transcription states, we identified breast cancer subtype specific patterns for repressive chromatin states (Additional file 14: Table S6). Distinct H3K27me3 signal patterns were observed, revealing subtype specificity similar to that in active chromatin states (Fig. 2d). The non-malignant, immortal cells had the greatest number of enriched/depleted repressive chromatin states relative to the breast cancer cell lines, which suggests that repressive states might serve as a pan breast cancer signature. Further functional analysis on associated gene sets did not reveal significantly enriched regulatory pathways, in contrast to the results for the active chromatin states. However, we observed one striking enrichment in the cluster of NOD-like signaling receptor genes, which display significantly increased H3K27me3 occupancy in all breast cancer cell lines but not in the normal immortalized cells (Additional file 15: Figure S7). NOD-like signaling receptors are involved in inflammation and immune responses, and have been described as master regulators in cancer development [8]. Our results are consistent with decreased expression of the NOD-like signaling receptor, NLRP3, in all subtypes of breast cancer relative to normal controls in the TCGA datasets (Additional file 13: Figure S6). The enrichment of repressive chromatin states on NOD-like gene family members in all breast cancer cells illustrates the regulatory potential for epigenetic silencing of cancer suppressive genes and also provides a potential marker for a pan breast cancer signature.

### TNBC specific chromatin signature

**Fig. 3:**
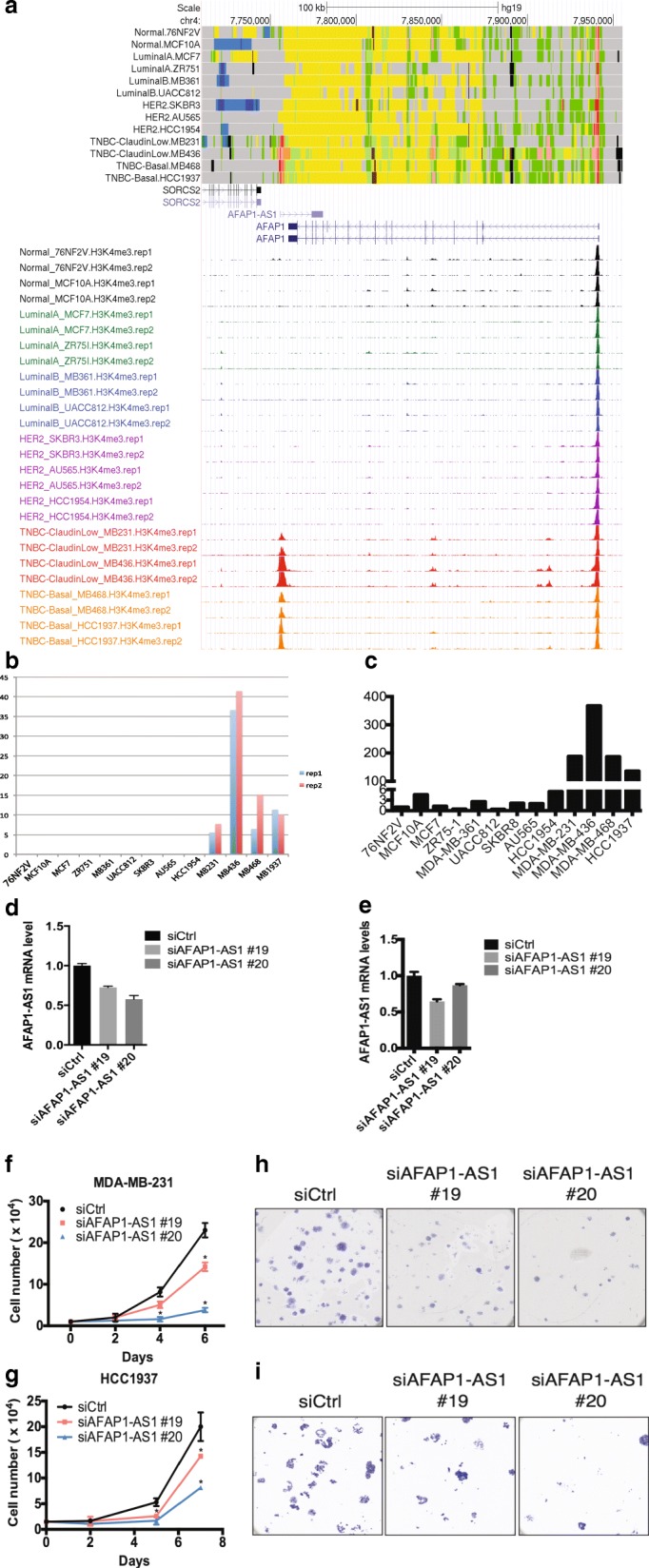
TNBC specific signatures: AFAP-AS1 transcription. **a** Screen shot of TNBC specific chromatin states and H3K4me3 signals on AFAP1-AS1 promoter and genebody. TNBC specific AFAP1-AS1 expression measured by (**b**) RNA-seq and (**c**) q-PCR. Depletion of AFAP1-AS1 in MB231 (**d**) and HCC1937 cells (**e**) inhibits cell proliferation (**f**, **g**), and colony formation (**h**, **i**) respectively

Close inspection of these chromatin states signatures also identified individual genes that displayed highly specific patterns across the breast cancer subtypes. For example, both active promoter and transcription signatures (Fig. 3a) and RNA-Seq (Fig. 3b) identified Actin Filament Associated Protein Antisense RNA 1 (AFAP1-AS1), an anti-sense long non-coding RNA (lncRNA), as a TNBC specific gene, marked by multiple active histone modifications, such as H3K4me3 and H3K79me2. Exclusive expression of AFAP-AS1 is in triple negative breast cancer cells was further confirmed by RT-qPCR (Fig. 3c).

Interestingly, although AFAP1-AS1 has not been linked to TNBC previously, it has been reported to be highly expressed and to predict poor prognosis in various types of cancers, including Barrett’s esophagus and esophageal adenocarcinoma [9, 10], pancreatic ductal adenocarcinoma [11], lung cancer [12, 13], nasopharyngeal carcinoma [14], hepatocellular carcinoma [15, 16], and colorectal cancer [17]. In Barrett’s esophagus adenocarcinoma, AFAP1-AS1 promoter DNA is largely hypomethylated, resulting in higher expression relative to matched normal tissues [9]. Interestingly, the protein coding counter part of AFAP1-AS1, AFAP1, is not a regulatory target for AFAP1-AS1 [9]. No direct targets for AFAP1-AS1 have been identified, but expression of the epithelial marker E-cadherin is repressed upon ectopic expression of AFAP1-AS1 whereas mesenchymal markers Vimentin, N-cadherin, Slug, Snail expression levels are elevated [11]. AFAP1-AS1, then, may promote proliferation, migration, or invasion of cancer cells by facilitating epithelial-mesenchymal transition (EMT). Consistent with these previous findings, our data indicate that AFAP1 does not show any subtype specificity in terms of histone modification occupancy and transcription levels (Fig. 3a, b), further indicating that AFAP1-AS1 does not directly regulate AFAP1 expression.

To determine if AFAP1-AS1 affects the growth or aggressiveness of TNBC cells, we depleted AFAP1-AS1 expression using two siRNAs targeting different regions of AFAP1-AS1 in two TNBC cell lines, MDA-MB-231 and HCC1937 (Fig. 3d, e). Limited knock down of AFAP1-AS1 (~ 25–50%), was sufficient to decrease proliferation (Fig. 3g, h) and inhibit colony formation (Fig. 3i, j) of both MDA-MB-231 cells and HCC1937 cells. The functions of AFAP1-AS1 are not yet clear, but the identification AFAP1-ASI as a TNBC specific gene through analysis of subtype specific chromatin states illustrates the power of our approach in identifying novel molecular targets for future development of TNBC therapies.

### NAA60/ZNF597 imprinting

Our analyses also identified loci that are specifically silenced in TNBC cells. One striking example is located at the bi-directional promoter of NAA60/ZNF597, which displays a complete loss of active promoter marks in both TNBC subtypes (Additional file 16: Figure S8). ZNF597 is further validated to have lower expression in TCGA basal patients compared with other breast cancer subtype patients (Additional file 13: Figure S6). NAA60 is a histone acetyltransferase that mediates several acetylation events in H4, including H4K20ac, H4K79ac and H4K91ac. The long isoform of NAA60 is subject to allele specific imprinting, while in contrasts, the shorter isoforms are bi-allelically expressed. The complete reduction of H3K4me3 at the long isoform promoter is consistent with bi-allelic silencing of the longer isoform. These results highlight the power of chromatin states signatures to not only predict transcriptional status but also to infer other potential epigenetic patterns, such as DNA methylation states.

## Discussion

The LONESTAR consortium data provide a comprehensive resource for histone modification profiles and transcription states across a novel collection of breast cancer cell lines that represent the 5 molecular subtypes of breast cancer. Integrated analyses of these data defined chromatin state landscapes across human breast cancer cell lines and identified subtype specific epigenetic signatures for major breast cancer subtypes. These epigenetic signatures revealed functional gene sets for each of the five breast cancer subtypes. Our results are consistent with previous breast cancer profiling studies, but also provide unique insights, such as discovery of AFAP1-AS1 (Fig. 3) and NAA60 (Additional file 16: Figure S8) as potential TNBC subtype specific genes. The chromatin state landscapes defined here in breast cancer cells also demonstrate the complexity of interactions between covalent histone modifications.

## Conclusions

Ultimately, these data may provide new clues to the etiology of the different breast cancer subtypes and new avenues for therapy development. We hope these data resources as well as our analyses will be broadly used in the breast cancer research community for mechanistic studies, biomarker discovery and precision therapy.

## Methods

### Experimental procedures

#### Cell cultures

All cell lines used for the Lonestar Consortium were purchased from ATCC (http://www.ncbi.nlm.nih.gov/pubmed/23722650):

Cell line ATCC ID.

76NF2V REF.

MCF10A CRL-10317.

MCF7 HTB-22.

ZR751 CRL-1500.

MB361 HTB-27.

UACC812 CRL-1897.

SKBR3 HTB-30.

AU565 CRL-2351.

HCC1954 CRL-2338.

MB231 CRM-HTB-26.

MB436 HTB-130.

MB468 HTB-132.

HCC1937 CRL-2336.

Cells were grown and cared for in the laboratory of Khandan Keyomarsi at the MD Anderson Cancer Center to ensure reproducibility and equity among all the labs in the consortium. RNA, protein isolates, chromatin extracts and nuclei preparations were performed in the cell culture core lab and distributed to the different member labs of the consortium for downstream experimentation. The two immortalized breast epithelial cell lines, MCF-10A and 76 N–F2V, were grown in D-Media and all other cell lines were grown in alpha media. All cells were grown in cell culture incubators at 37oC with 6.5% CO2. For D-media, the following components are added to an equal mixture of alpha-MEM and Ham’s F12 base media; 0.1 M HEPES, 2 mM L-glutamine, 1% FBS, 0.035 mg/ml of Bovine Pituitary Extract, 0.01 mM Ascorbic Acid, 2 nM β-estradiol, 2.5 ng/ml Sodium Selenite, 10 nM Triiodothryonine, Ethanolamine, 1 μg/ml Insulin, 1 ng/ml Hydrocortisone, 0.1 mM Phosphoethanolamine, 0.01 mg/ml Transferrin, 12.5 ng/ml Epidermal Growth Factor, 1% Penn/Strep. For alpha media the following components are added to α-MEM base media; 0.1 M HEPES, 10% Fetal Calf Serum, 1% non-essential amino acids, 2 mM L-glutamine, 1% Sodium Pyruvate, 1 μg/ml insulin, 1 ng/ml Hydrocortisone, 12.5 ng/ml Epidermal Growth Factor, 1% Penn/Strep.

### Small interfering RNA transfection

When cells reached 60% confluence, two siRNAs targeting AFAP1-AS1 (ThermoFisher Scientific, Cat. # 4390771, n262319 and n262320) and a control siRNA were transfected separately using Lipofectamine® 2000 (Life Technologies Cat. # 11668019) following manufacturer’s instructions. Six to eight hours after transfection, the medium was changed to normal culture medium as described above.

### RNA extraction, reverse transcription and quantitative real-time PCR (qRT-PCR)

Total RNAs were isolated using an RNeasy Mini kit (Qiagen, Cat. # 74104), following the manufacturer’s recommended procedure. 1 μg of RNA was reverse-transcribed to cDNA using a SuperScript VILO cDNA Synthesis kit (ThermoFisher Scientific, Cat. # 11754050) following the manufacturer’s procedure. 10 ng of cDNA was used for one qRT-PCR reaction with three technical replicates.

### Cell proliferation assay

MDA-MB-231, HCC1937 cells were transfected with control siRNA, or siRNA targeting AFAP1-AS1. After 48 h, 15,000 cells were seeded into each well of 12 well plate with three replicates for each sample and each day of counting. At indicated day, cell numbers were counted using hemocytometer.

### Colony formation assay

MDA-MB-231, HCC1937 cells were transfected with control siRNA, or siRNA targeting AFAP1-AS1. After 48 h, 300 cells for each sample were put into single well of a 6 well plate, with three replicates, and incubated at 37 °C with 5% CO_2_. 10 to 15 days later, cells were washed with PBS once and fixed with Fixation buffer (Acetic acid/methanol, 1:7 *v*/v) for 5 min. Then cells were stained with 0.5% crystal violet for 2 h to overnight, washed, dried, and imaged.

### Chromatin immunoprecipitation and library preparation

The following antibodies were used qPCR- and sequencing-based ChIP assays in the amounts specified:

**Table:** 

Modification or Factor	Amount per IP	Company	Catalog No
H3K4me1	5 μg	Abcam	ab8895
H3K4me3	5 μg	Abcam	ab8580
H3K9ac,	5 μg	EMD Millipore	07–352
H3K9me3	5 μg	Abcam	ab8898
H3K27ac	5 μg	Abcam	ab4729
H3K27me3	5 μg	Millipore	07–449
H3K36me3	5 μg	Abcam	ab9050
H3K79me2	5 μg	Abcam	ab3594
H2BK120ub1	5 μg	Millipore	05–1312
H3K23ac	5 μg	Millipore	07–355
H4K8ac	5 μg	Millipore	07–328

Cells were grown to ~ 70–80% confluence, cross-linked with 1% formaldehyde for 10 min at 37 °C, and quenched in 125 mM glycine for 5 min at 4 °C. The cells were then collected and lysed in Farnham Lysis Buffer [5 mM PIPES pH 8.0, 85 mM KCl, 0.5% NP-40, 1 mM DTT, and 1× protease inhibitor cocktail (Sigma-Aldrich)]. The crude nuclear pellet was collected by centrifugation, resuspended in lysis buffer (1% SDS, 10 mM EDTA, 50 mM Tris•HCl pH 7.9, 1 mM DTT, and 1× protease inhibitor cocktail), and incubated on ice for 10 min. The chromatin was sheared by sonication at 4 °C using a Bioruptor 300 at the highest setting for fifteen 1-min cycles of 30 s on and 30 s off to generate chromatin fragments of ~ 200–400 bp in length. The soluble chromatin was diluted 1:10 with dilution buffer (20 mM Tris•HCl, pH 7.9, 0.5% Triton X-100, 2 mM EDTA, 150 mM NaCl, 1 mM DTT and 1× protease inhibitor cocktail) and pre-cleared with protein A agarose beads. Five percent of the material was removed and saved as input, and the rest of the pre-cleared supernatant was incubated overnight at 4 °C with the antibody of interest and a non-specific IgG control antibody.

The following day, the immune complexes were collected by adding protein A agarose beads and incubating for 2 h at 4 °C. The immunoprecipitated material was washed once with low salt wash buffer [20 mM Tris•HCl pH 7.9, 2 mM EDTA, 125 mM NaCl, 0.05% SDS, 1% Triton X-100, and 1× protease inhibitor cocktail], once with high-salt wash buffer (20 mM Tris•HCl pH 7.9, 2 mM EDTA, 500 mM NaCl, 0.05% SDS, 1% Triton X-100, and 1× protease inhibitor cocktail), once with LiCl wash buffer (10 mM Tris•HCl pH 7.9, 1 mM EDTA, 250 mM LiCl, 1% NP-40, 1% sodium deoxycholate, and 1× protease inhibitor cocktail), and twice with Tris-EDTA (TE) containing 1× protease inhibitor cocktail. The immunoprecipitated material was eluted at room temperature in elution buffer (100 mM NaHCO_3_, 1% SDS), and the crosslinks were reversed by adding 100 mM NaCl with incubation at 65 °C overnight. The eluted material was then digested with proteinase K and RNase H to remove protein and RNA, respectively, and the enriched genomic DNA was extracted with phenol:chloroform:isoamyl alcohol followed by ethanol precipitation. The ChIPed DNA was dissolved in water and analyzed by qPCR using the enhancer- or gene-specific primers.

ChIP libraries were prepared using a modified Kapa LTP Library Preparation kit (KAPA Biosystems, cat# KK8232) for Illumina Platforms. Ten ng of sheared DNA was used to repair the ends of the damaged fragments using a proprietary master mix. The resulted blunted fragments were 3′ A-tailed using a proprietary mixture of enzymes to allow ligation to the specific NexTflex adaptors from Bioo Scientific (Bioo Scientific, cat# 514102). Each of the steps (i.e., end repair, 3’A tailing, and adaptor ligation) was followed by column clean up (Qiagen, cat# 28204). After adapter ligation, DNA enrichment was performed using Kapa HiFi Hot Start Ready PCR mix, and a cocktail of primers (1 cycle at 98 °C for 45 s; 4 cycles at 98 °C for 15 s, 60 °C for 30 s, and 72 °C for 30 s; and 1 cycle at 72 °C for 1 min), and purified with AmpureXP beads (Beckman Coulter, cat# A63881). The quality of the final libraries was assessed using a 2200 TapeStation (Agilent Technologies). The libraries were quantified using a Kapa Library Quantification Kit (KAPA Biosystems, cat# KK4933) and loaded in a flow cell for cluster generation using the Illumina cBOT (Illumina) at final concentration of 10 pM.

### ChIP-seq data processing

ChIP-seq libraries were sequenced on Hi-seq2500, with 50 bp single-end raw reads. For each cell line, two replicates were sequenced in the 8 chosen histone modifications (Fig. 1a). Three additional histone modifications, H2BK120ub1, H3K23ac and H4K8ac, were also sequenced for each cell line, with no replicates. ChIP-seq raw reads were mapped to hg19 reference genome using bowtie v1.0,0 [18], with command line options “-v 1 -r --best --strata -m 1” to allow up to one mismatch per read. To eliminate the read length variations in different sequencing batches, all reads were clipped to the first 36 bp. The mapped reads were complied into whole genome WIG profile using MACS 1.3.7 [19], with the fragment size set to the 200 bp and peak calling threshold set at *p*-value = 1e-8. To reduce the signal strength variation due to sequencing depth difference, we subsampled to a maximum of 20 million reads per replicates per sample. The reproducibility between replicates was evaluated by the Pearson correlation of peak intensities (Additional file 2: Table S1). Hierarchical clustering is performed on 2000 peaks randomly sampled from the union of peak lists called from all ChIP-seq samples. The max heights of the peak intensity were used and the values were standardized for each peak across cells in the clustering. The hierarchical clustering were performed using MeV 4.8.0 [20].

### RNA-seq data processing

RNA-seq reads were mapped to hg19 reference genome and transcriptome using tophat v2.0.10 [21]. The guide transcriptome annotation GTF file is Ensembl gene annotation downloaded from genome.ucsc.edu. The reads count for each Ensembl gene (Additional file 17: Table S10) was estimated using HTseq v0.6.1p2 [22], with option “-s no -m intersection-nonempty”. The FPKM values were calculated by normalizing the read count by the total reads number in millions and gene length in kilo base pairs.

### Chromatin state model

For each histone mark and each cell line, the alignment bed files of two replicates were pooled. The reads were extended to 200 bp from 5′ to 3′ or 3′ to 5′ direction for positive and negative strands alignments respectively. We used ChromHMM v1.1.12 [5] to binarize the ChIP-seq signals with default parameters and build the chromatin state model at 200 bp resolution (−b 200), on all cell line samples for 5 major histone marks: H3K4me1, H3K4me3, H3K9me3, H3K27me3 and H3K36me3. We trained 11 ChromHMM models, covering from 10 to 20 total chromatin states, and decided to choose a 13-states model that best captures the combinatorial patterns between the histone marks. We also trained models using all 8 histone marks using same procedure, and the extended model (Additional file 5: Figure S3) identified 15 chromatin states that largely overlap with the chromatin states defined by the 5 core histone modification model, with additional states representing the 5′ or 3′ end of active transcription units or broad flanking regions of active promoters. (Fig. 1b). Therefore we used the five histone mark model in our subsequent analyses. We followed the method used by NIH roadmap epigenetics consortium to evaluate the robustness of the 13 chromatin-states model jointly-trained on all cell lines by comparing it with models independently trained on individual cell lines [4]. The hierarchical clustering of the emission parameters (Additional file 18: Table S3) of all trained ChromHMM models indicates that the jointly-trained model on all cell lines can be reproduced by independently-trained model (Additional file 4: Figure S2A).

### Subtype specific signature identification

We first identify all genomic regions that have same within-subtype chromatin states and different between-subtype chromatin states. This is done by detecting have same chromatin states in cell lines of the same subtype, and filter out the regions that have same chromatin states in all subtypes. The subtype specific chromatin state signature is defined by the chromatin states that are uniquely present or absent in the subtype. The pan breast cancer signature is defined by comparing normal like cells with breast cancer cells, and the triple negative signature is defined as comparing TNBC-basal/claudinLow cells with Luminal/HER2 positive cells. We further characterized the subtype pattern by their representative histone mark signals. We profiled the max H3K36me3 peak heights within the genebody for TxAct and TxFlk states, max H3K27me3 peak height within Genebody for RepPC and WkRep states, max H3K4me3 signals within 1Kbp of TSS for PrAct, PrFlk and PrBiv states, max H3K4me1 peak heights within 1Kbp of EhAct, EhGen and EhBiv genomic regions, max H3K9me3 peak heights within Genebody for Htchr states.

## Additional files

Additional file 1: **Figure S1.** Enrichment of histone modification ChIP-seq signals pooled from all sampled in **(A)** transcription start sites, **(B)** gene bodies, **(C)** enhancer peaks. **(D)** Genomic distribution of histone modification ChIP-seq tags. **(E)** Hierarchical clustering of histone modification ChIP-seq peak signals. (PDF 936 kb)

Additional file 2: **Table S1**. ChIP-seq sample alignment summary. (XLSX 66 kb)

Additional file 3: **Table S2**. ChIP-seq sample replicates correlation. (XLSX 61 kb)

Additional file 4: **Figure S2**. **(A)** Clustering of chromatin states model learned on individual cells showing same enrichment pattern that can recover the chromatin state jointly learned using all 13 cells. **(B)** RNA-seq expression levels for genes associated with different chromatin states. **(C)** Cumulative fractions of chromatin state counts versus number of samples. Larger area under curve indicates more variability across breast cancer cells. (PDF 1549 kb)

Additional file 5: **Figure S3.** ChromHMM model of 15 chromtain states defined by all 8 histone modifications. (PDF 358 kb)

Additional file 6: **Figure S4**. Unsupervised clustering of histone modification occupancy in highly variable regions (A, C, E, G, I, K, M, O) and enriched genomic regions (promoters: B, D, N, enhancers: F, L and gene bodies: H, J, P)., showing subtype specificity and reproducibility between replicates. (PDF 2936 kb)

Additional file 7: **Table S5**. List of genes with subtype specific active enhancer states. (XLS 212 kb)

Additional file 8: **Figure S5.** Spearman correlation of H3K36me3 occupancy and gene expression levels in all samples. (PDF 489 kb)

Additional file 9: **Table S4**. List of genes with subtype specific active transcription states. (XLS 44 kb)

Additional file 10: **Table S7**. Subtype gene signatures of active transcription states and active transcription flanking states. (XLSX 23 kb)

Additional file 11: **Table S8**. Significant pathways of subtype gene signatures of active transcription states and active transcription flanking states. (XLSX 18 kb)

Additional file 12: **Table S9** Significant upstream regulators of subtype gene signatures of active transcription states and active transcription flanking states. (XLSX 16 kb)

Additional file 13: **Figure S6.** Subtype expression patterns of TCGA breast cancer samples. (PDF 359 kb)

Additional file 14: **Table S6**. List of genes with subtype specific repressive polycomb domain states. (XLS 10 kb)

Additional file 15: **Figure S7.** Chromatin state landscape of depleted H3K27me3 signals at NLRP gene cluster in normal-like celllines. (PDF 595 kb)

Additional file 16: **Figure S8.** Chromatin state landscapes of depleted H3K4me3 signals in the bi-directional promoter of NAA60/ZNF597 in TNBC subtype celllines. (PDF 328 kb)

Additional file 17: **Table S10**. RNA-seq gene expression read count. (XLSX 8751 kb)

Additional file 18: **Table S3**. Emission probability and transition probability of Chromatin States defined by ChromHMM. (XLSX 46 kb)

## Abbreviations

AFAP1-AS1: Actin filament associated protein antisense RNA 1
CHDH: Choline dehydrogenase
ChIP-Seq: Chromatin immunoprecipitation sequencing
EhAct: active enhancers in intergenic regions
EhBiv: bivalent enhancers
EhGen: active enhancers in genic regions
EMT: epithelial-mesenchymal transition
ENCODE: Encyclopedia of DNA Elements
ER: Estrogen receptor
eRNA: enhancer RNA
GRO-seq: Global run-on sequencing
HER2: human epidermal growth factor receptor 2
Htchr: heterochromatin
lncRNA: long non-coding RNA
LONESTAR: Lonestar Oncology Network for Epigenetics Therapy And Research
PrAct: active promoters
PrBiv: poised bivalent promoters
PrFlk: promoter flanking regions
qRT-PCR: quantitative real-time PCR
QsLow: quiescent/low signal regions
RepPC: strong repressive polycomb domains
RNA-Seq: RNA sequencing
RpZNF: repeats/ZNF gene clusters
TCGA: The cancer genome atlas
TNBC: Triple-negative breast cancer
TSS: transcription start site
TxAct: active transcription units
TxFlk: flanking regions of active transcription units
WkREP: weak repressive polycomb domains
