# Supplementary figures and images for: Histone modification profiling in breast cancer cell lines highlights commonalities and differences among subtypes
Source: BMC Genomics. 2018 Feb 20;19:150. doi: 10.1186/s12864-018-4533-0 (PMC5819162; doi:10.1186/s12864-018-4533-0)

Figure S1

A

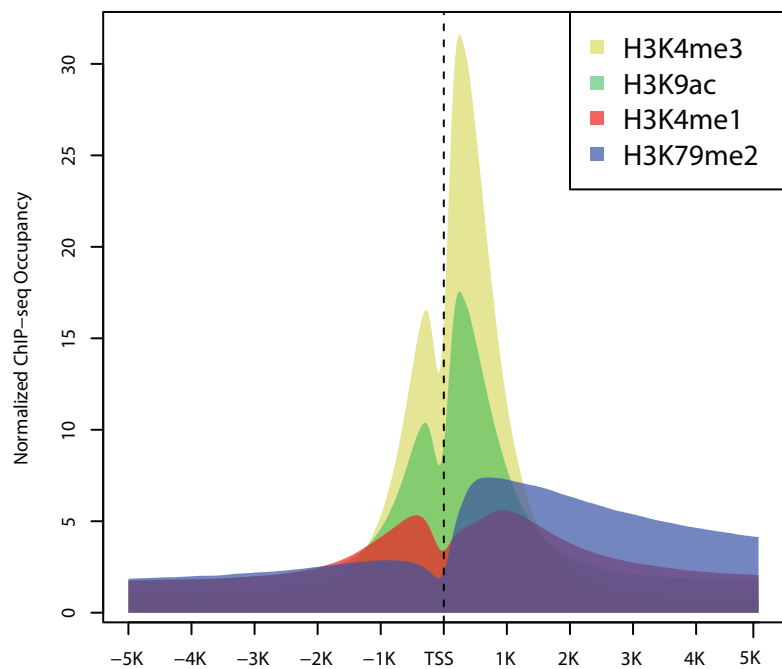

B

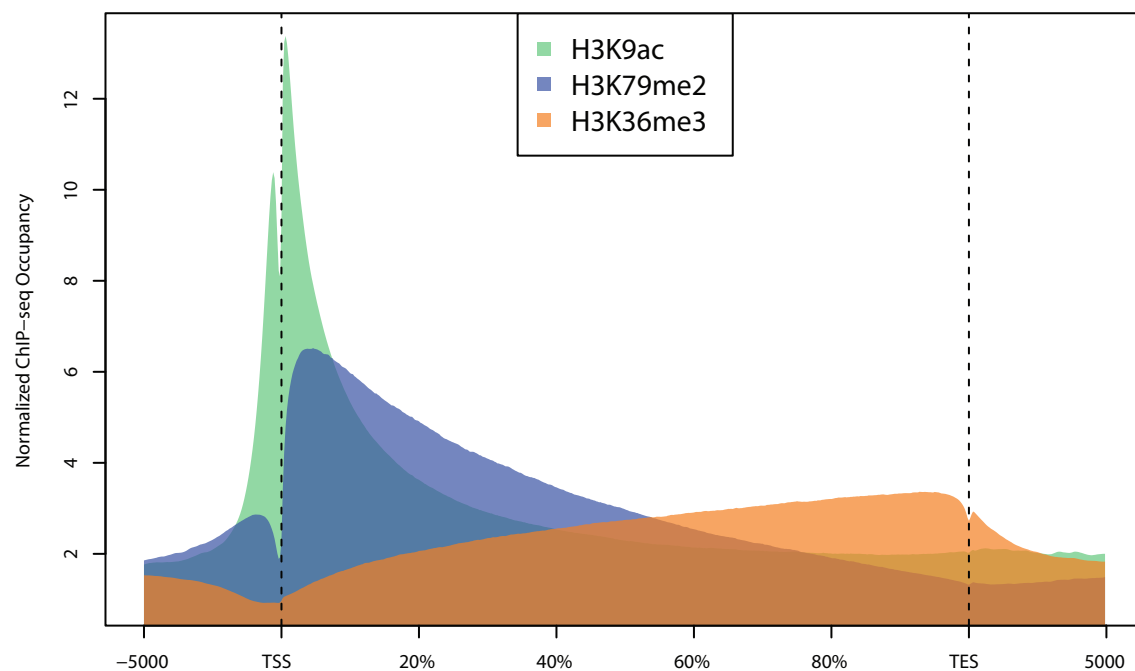

C

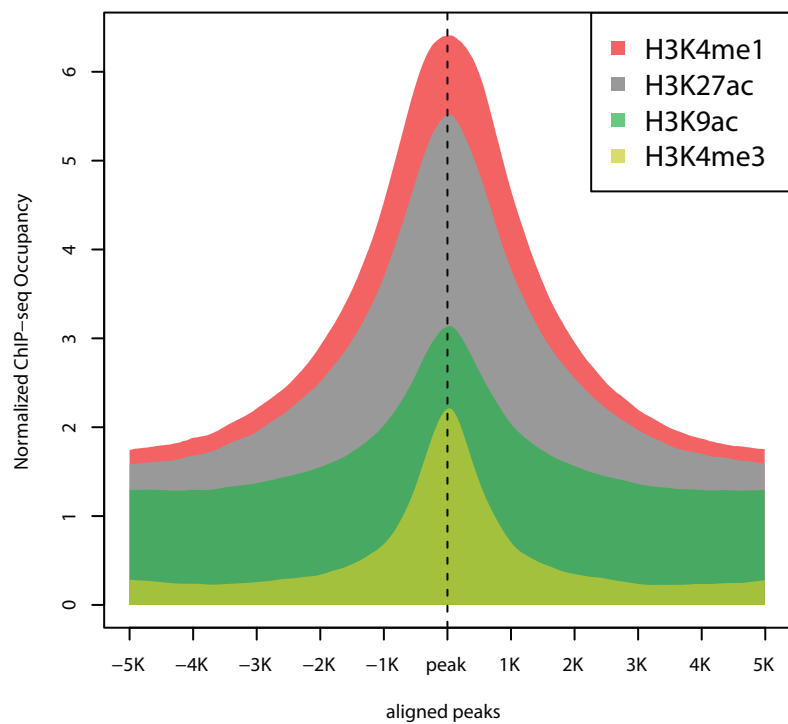

D

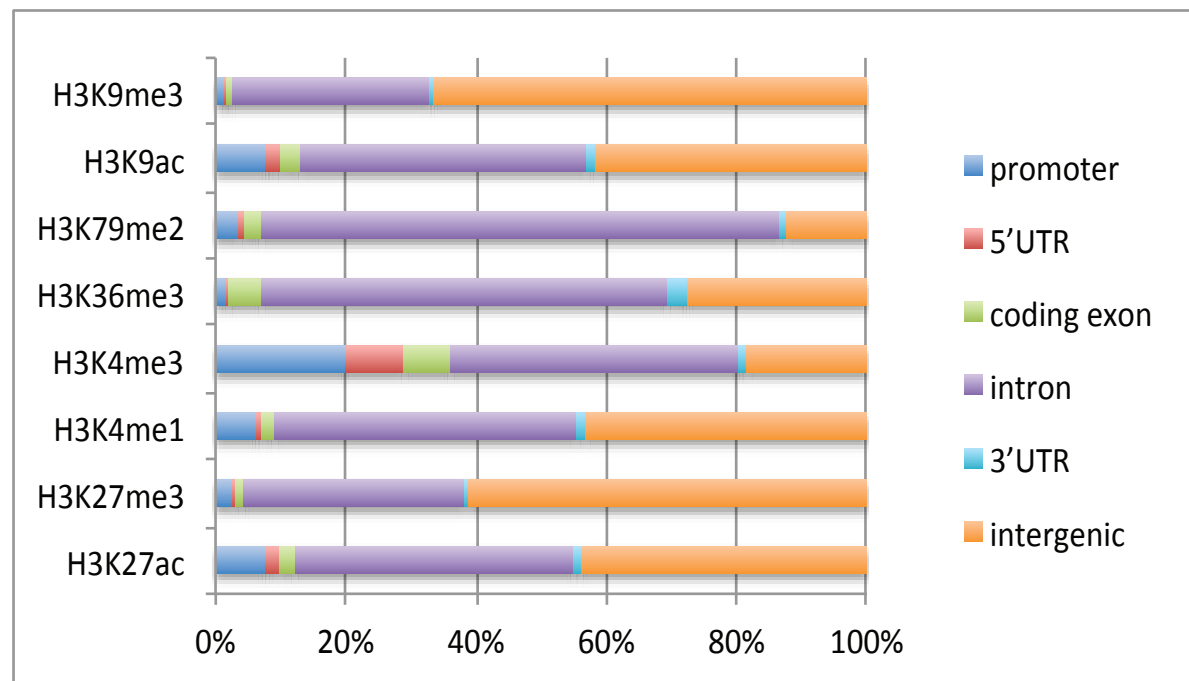

Supplement: Supplementary file 1 — Figure S1. Enrichment of histone modification ChIP-seq signals pooled from all sampled in (A) transcription start sites, (B) gene bodies, (C) enhancer peaks. (D) Genomic distribution of histone modification ChIP-seq tags. (E) Hierarchical clustering of histone modification ChIP-seq peak signals. (PDF 936 kb) [file 12864_2018_4533_MOESM1_ESM.pdf]

Figure S2

A

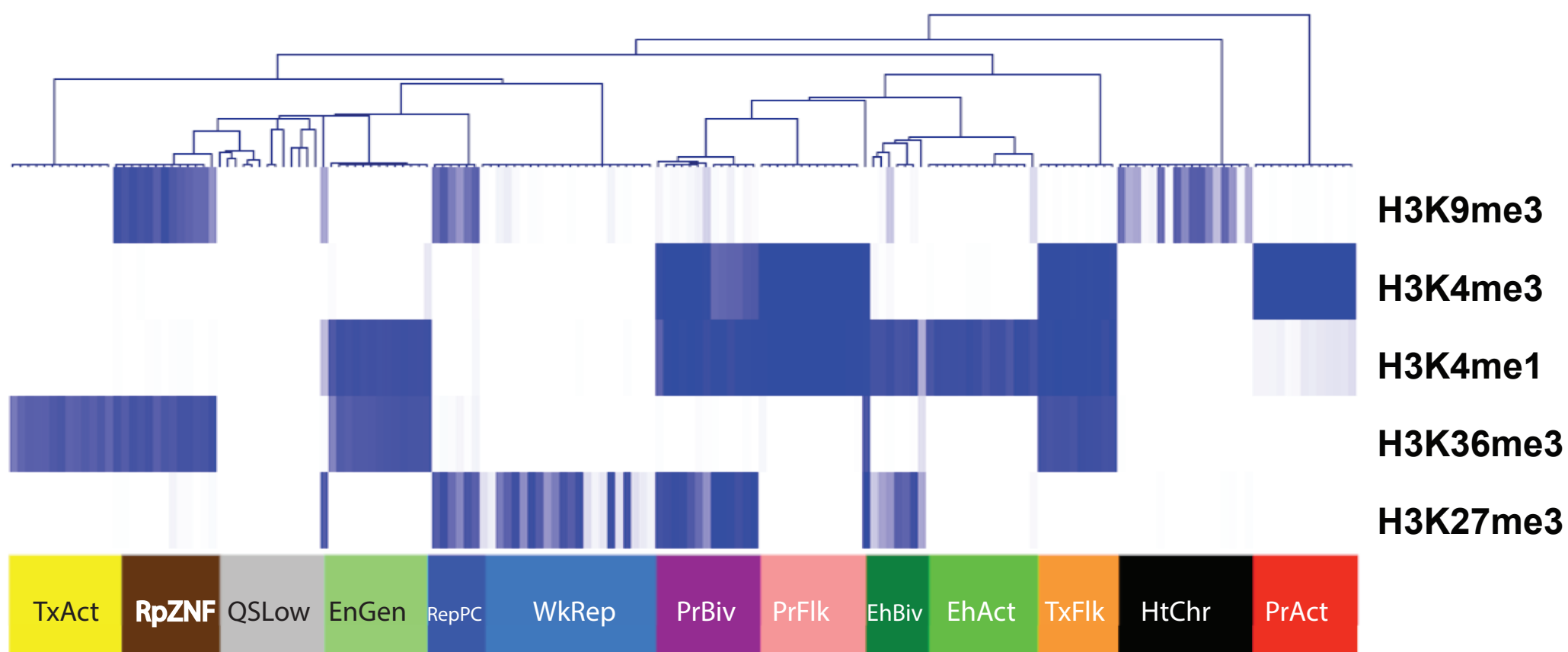

B

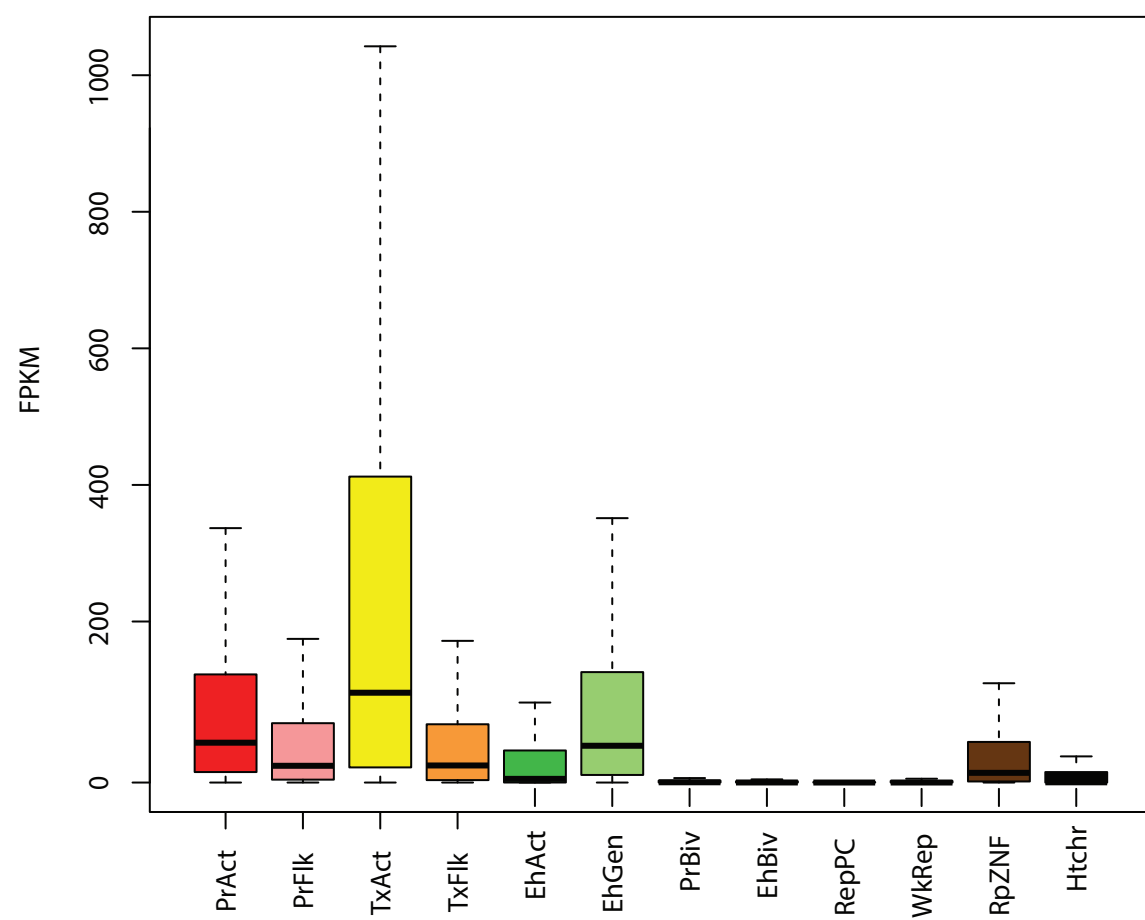

C

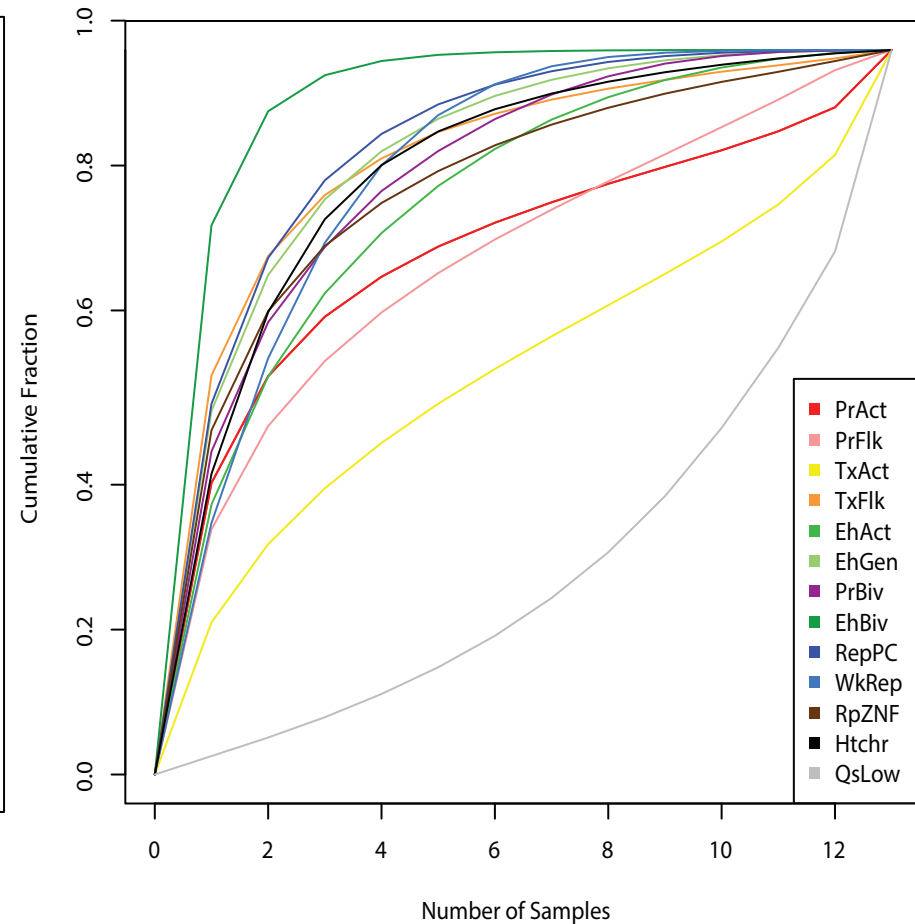

Supplement: Supplementary file 4 — Figure S2. (A) Clustering of chromatin states model learned on individual cells showing same enrichment pattern that can recover the chromatin state jointly learned using all 13 cells. (B) RNA-seq expression levels for genes associated with different chromatin states. (C) Cumulative fractions of chromatin state counts versus number of samples. Larger area under curve indicates more variability across breast cancer cells. (PDF 1549 kb) [file 12864_2018_4533_MOESM4_ESM.pdf]

Figure S5

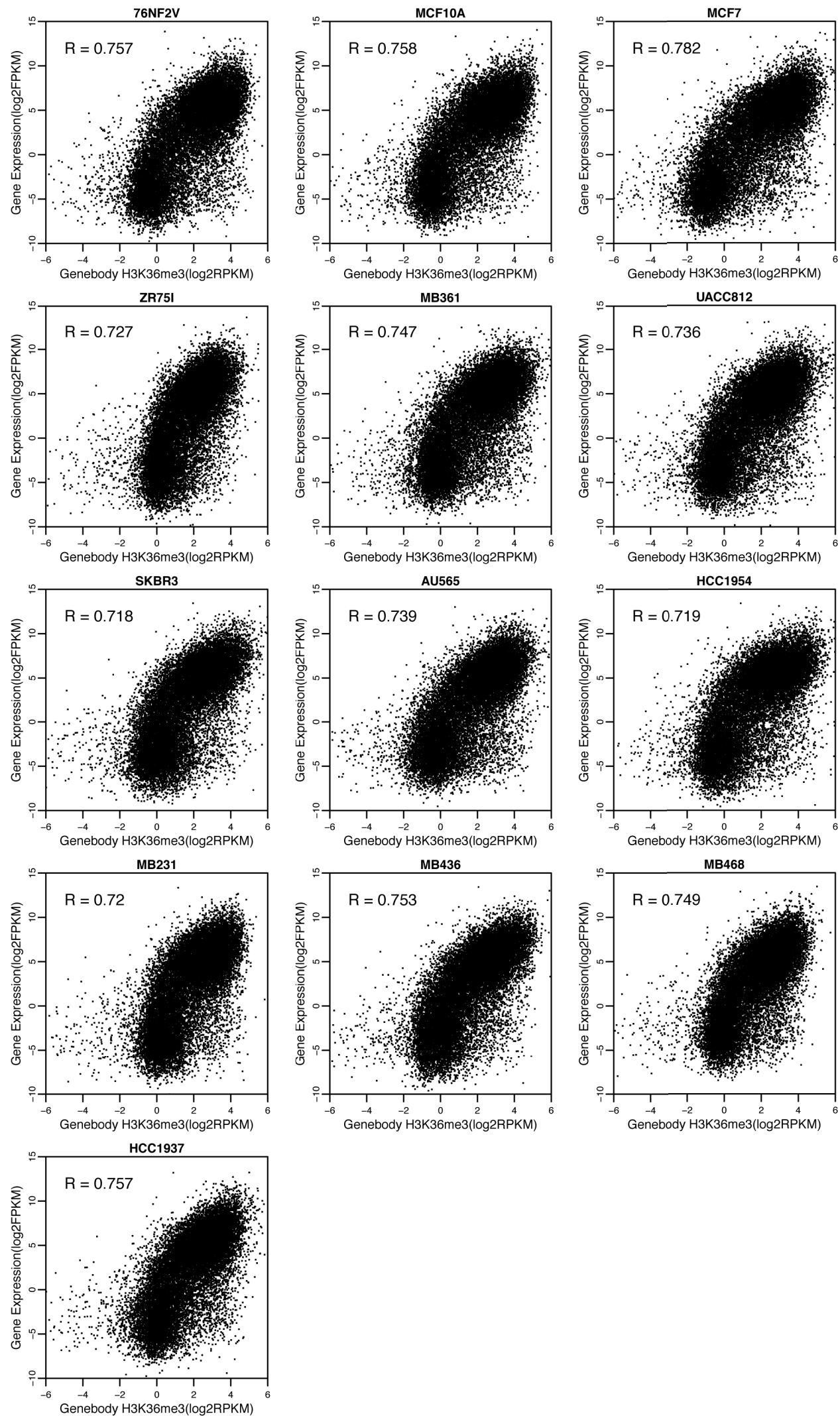

Supplement: Supplementary file 8 — Figure S5. Spearman correlation of H3K36me3 occupancy and gene expression levels in all samples. (PDF 489 kb) [file 12864_2018_4533_MOESM8_ESM.pdf]

Figure S6

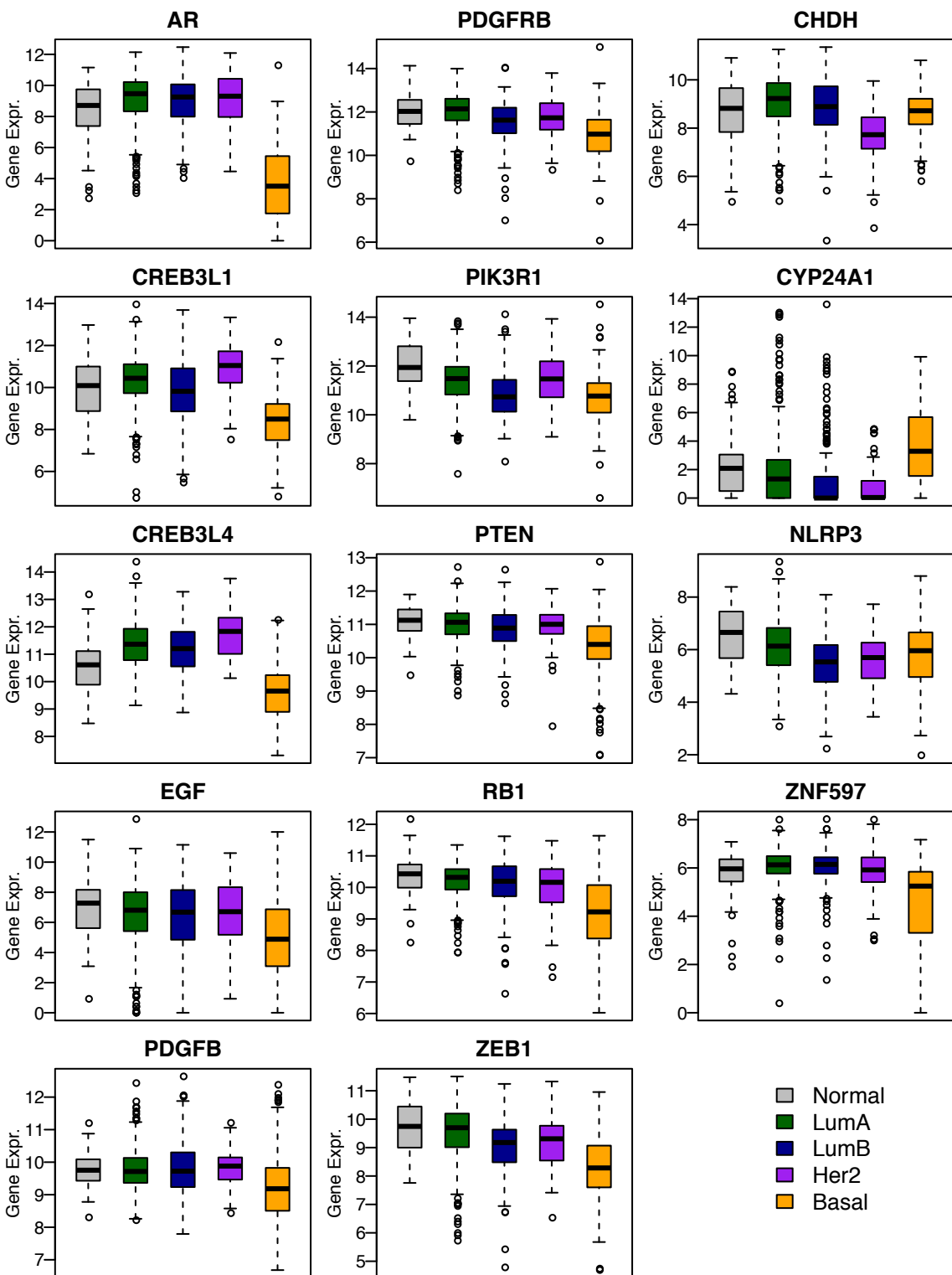

Supplement: Supplementary file 13 — Figure S6. Subtype expression patterns of TCGA breast cancer samples. (PDF 359 kb) [file 12864_2018_4533_MOESM13_ESM.pdf]

Figure S7

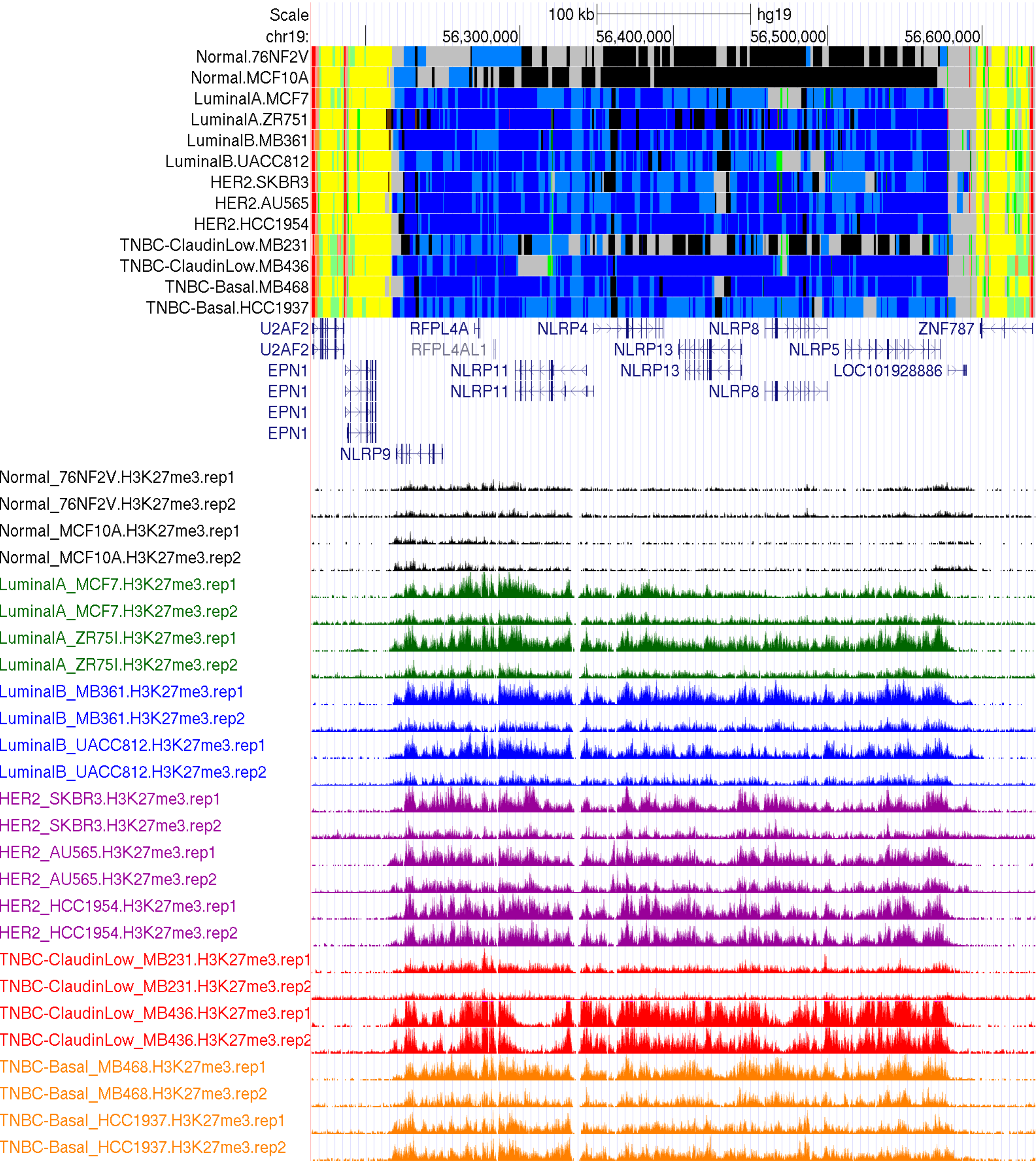

Supplement: Supplementary file 15 — Figure S7. Chromatin state landscape of depleted H3K27me3 signals at NLRP gene cluster in normal-like celllines. (PDF 595 kb) [file 12864_2018_4533_MOESM15_ESM.pdf]

Figure S8

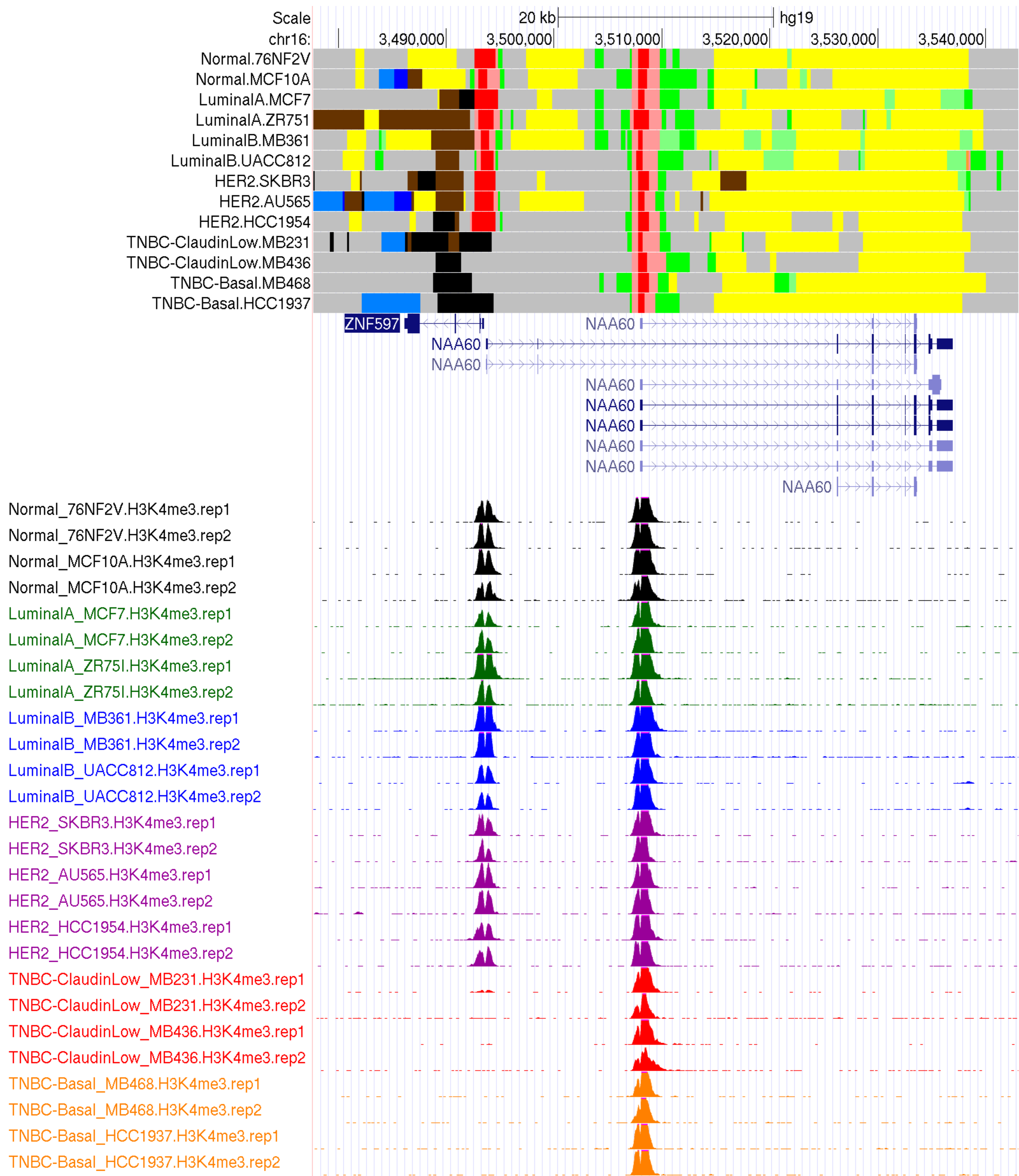

Supplement: Supplementary file 16 — Figure S8. Chromatin state landscapes of depleted H3K4me3 signals in the bi-directional promoter of NAA60/ZNF597 in TNBC subtype celllines. (PDF 328 kb) [file 12864_2018_4533_MOESM16_ESM.pdf]
