# Supplementary material for: Histone modification profiling in breast cancer cell lines highlights commonalities and differences among subtypes
Source: BMC Genomics. 2018 Feb 20;19:150. doi: 10.1186/s12864-018-4533-0 (PMC5819162; doi:10.1186/s12864-018-4533-0)

Figure S3

Chromatin State	Abbr.
Active Promoter	PrAct
Active Promoter Flanking	PrFlk
Active Promoter Broad	PrBrd
Active Promoter 3' Flanking	PrFlk3
Active Transcription	TxAct
Active Transcription 5'	TxAct5
Active Transcription 3'	TxAct3
Active Enhancers	EhAct
Active Genic Enhancer	EhGen
Active Enhancers Flanking	EhFlk
Bivalent Promoter	PrBiv
Repressive Polycomb Domain	RepPC
Weak Transcription	TxWk
Heterochromatin	Htchr
Quiescent State / Low Signals	QsLow

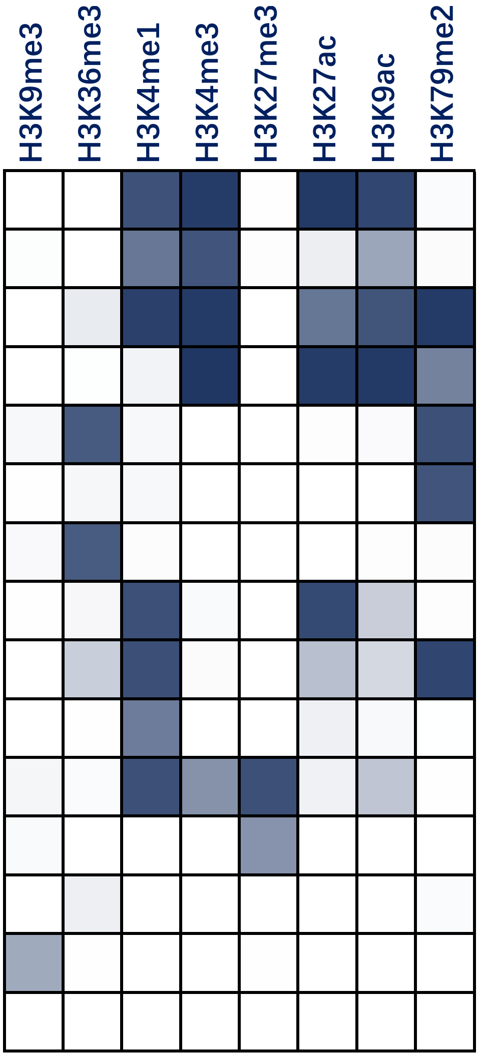

Supplement: Supplementary file 5 — Figure S3. ChromHMM model of 15 chromtain states defined by all 8 histone modifications. (PDF 358 kb) [file 12864_2018_4533_MOESM5_ESM.pdf]
