# Supplementary material for: Histone modification profiling in breast cancer cell lines highlights commonalities and differences among subtypes
Source: BMC Genomics. 2018 Feb 20;19:150. doi: 10.1186/s12864-018-4533-0 (PMC5819162; doi:10.1186/s12864-018-4533-0)
Supplement: Supplementary file 6 — Figure S4. Unsupervised clustering of histone modification occupancy in highly variable regions (A, C, E, G, I, K, M, O) and enriched genomic regions (promoters: B, D, N, enhancers: F, L and gene bodies: H, J, P)., showing subtype specificity and reproducibility between replicates. (PDF 2936 kb) [file 12864_2018_4533_MOESM6_ESM.pdf]

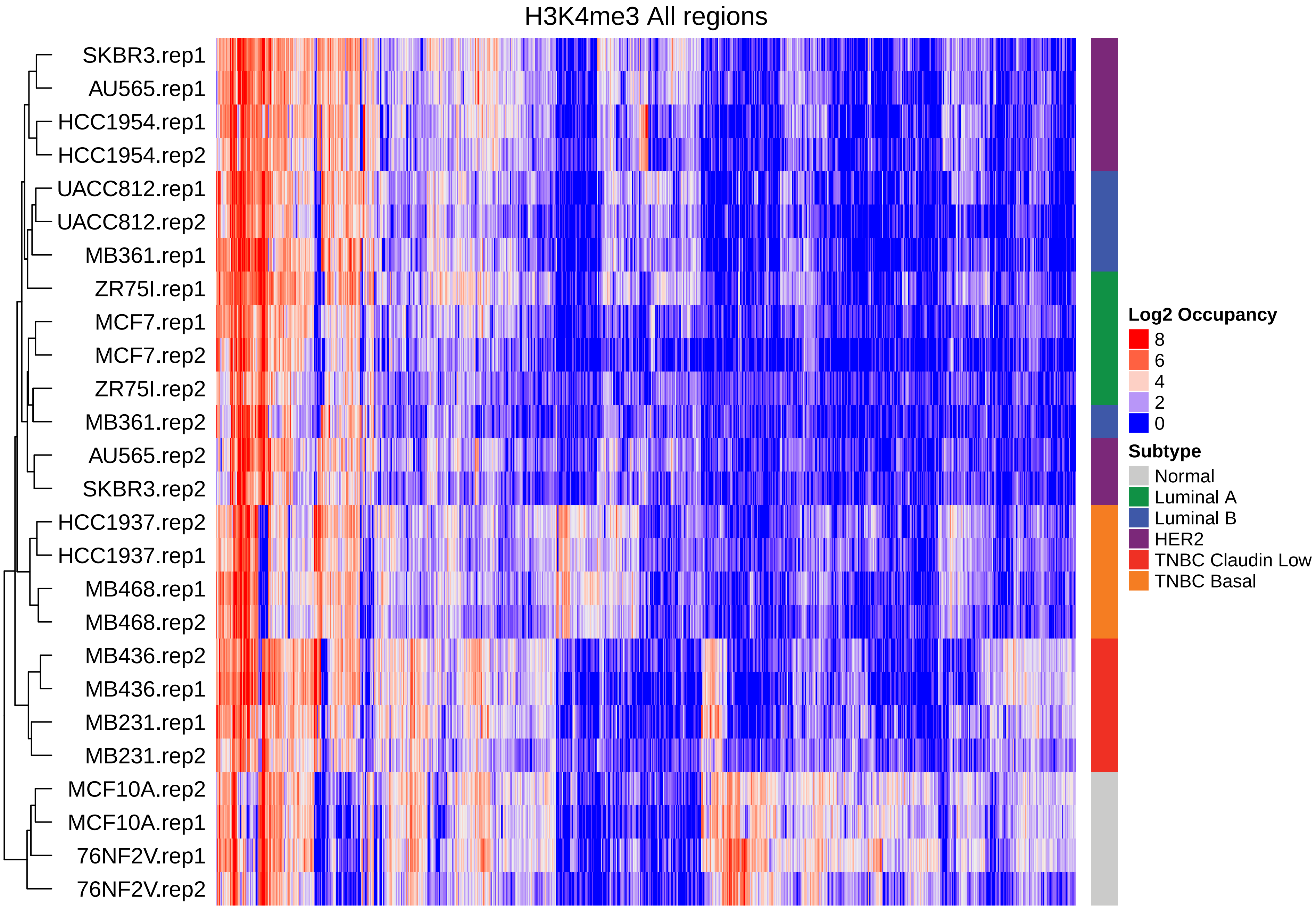

H3K4me3 All regions
SKBR3.rep1
AU565.rep1
HCC1954.rep1
HCC1954.rep2
UACC812.rep1
UACC812.rep2
MB361.rep1
ZR75I.rep1
MCF7.rep1
MCF7.rep2
ZR75I.rep2
MB361.rep2
AU565.rep2
SKBR3.rep2
HCC1937.rep2
HCC1937.rep1
MB468.rep1
MB468.rep2
MB436.rep2
MB436.rep1
MB231.rep1
MB231.rep2
MCF10A.rep2
MCF10A.rep1
76NF2V.rep1
76NF2V.rep2
Log2 Occupancy
8
6
4
2
0
Subtype
Normal
Luminal A
Luminal B
HER2
TNBC Claudin Low
TNBC Basal

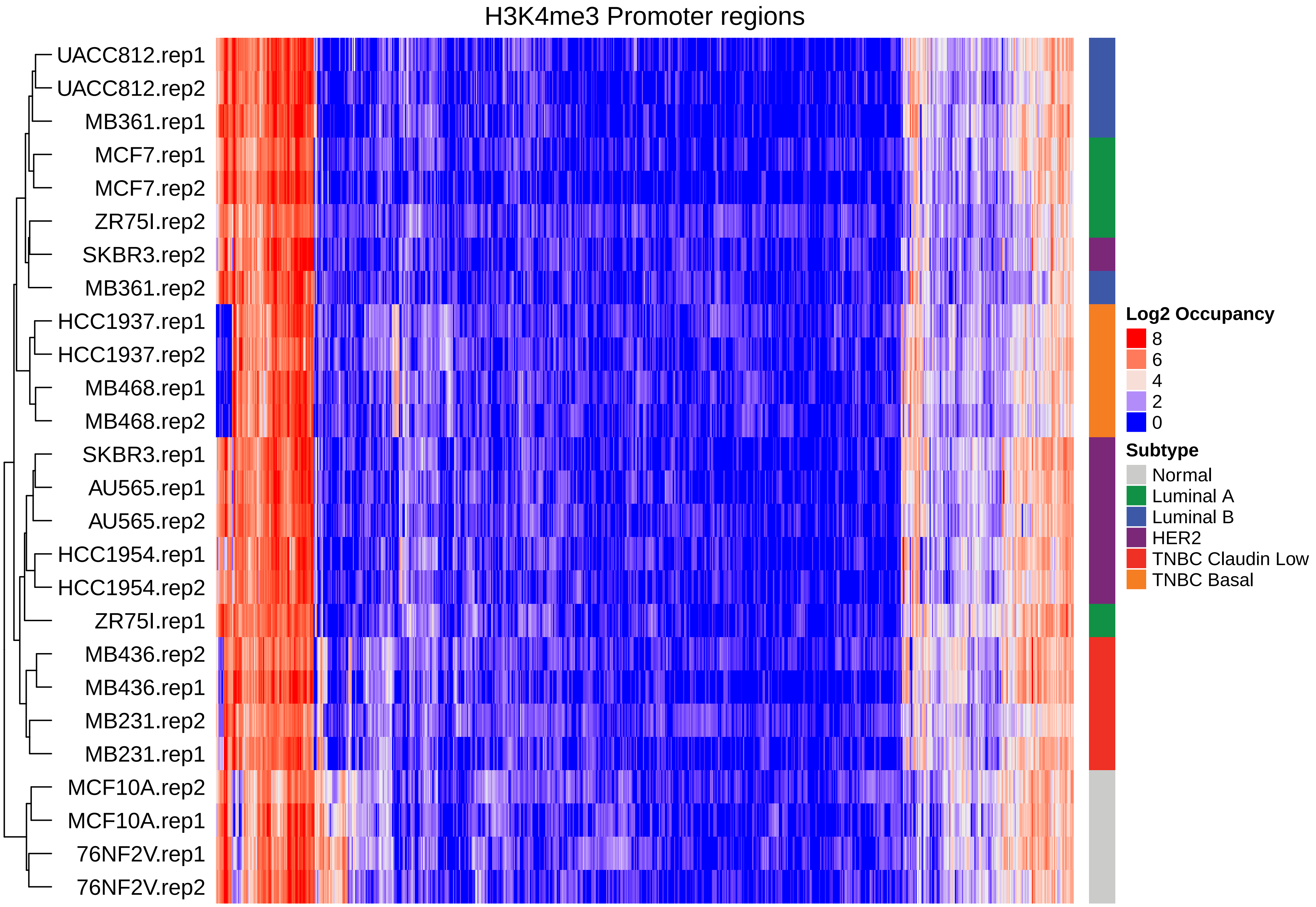

H3K4me3 Promoter regions
UACC812.rep1
UACC812.rep2
MB361.rep1
MCF7.rep1
MCF7.rep2
ZR75I.rep2
SKBR3.rep2
MB361.rep2
HCC1937.rep1
HCC1937.rep2
MB468.rep1
MB468.rep2
SKBR3.rep1
AU565.rep1
AU565.rep2
HCC1954.rep1
HCC1954.rep2
ZR75I.rep1
MB436.rep2
MB436.rep1
MB231.rep2
MB231.rep1
MCF10A.rep2
MCF10A.rep1
76NF2V.rep1
76NF2V.rep2
Log2 Occupancy
8
6
4
2
0
Subtype
Normal
Luminal A
Luminal B
HER2
TNBC Claudin Low
TNBC Basal

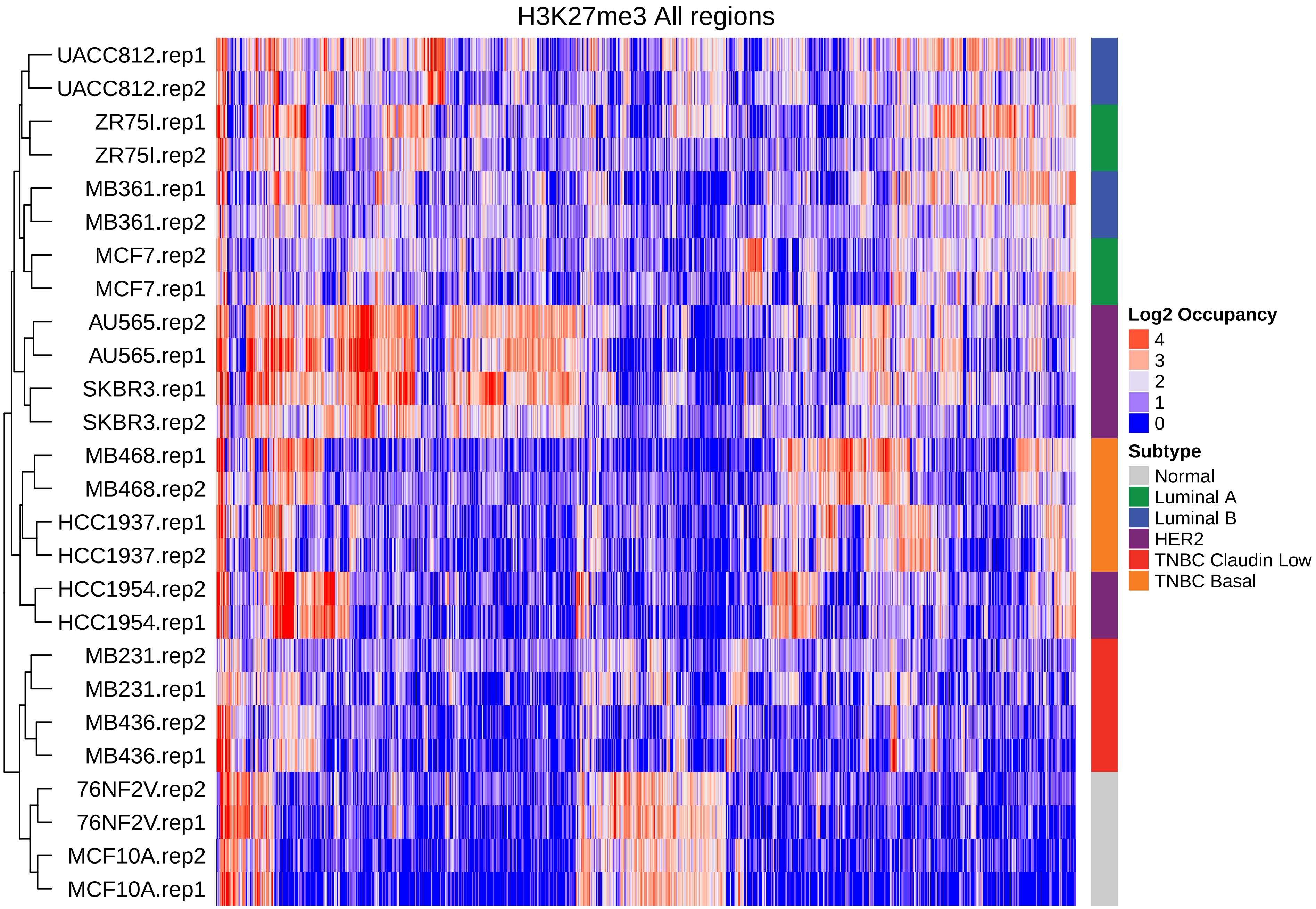
H3K27me3 All regions
UACC812.rep1
UACC812.rep2
ZR75I.rep1
ZR75I.rep2
MB361.rep1
MB361.rep2
MCF7.rep2
MCF7.rep1
AU565.rep2
AU565.rep1
SKBR3.rep1
SKBR3.rep2
MB468.rep1
MB468.rep2
HCC1937.rep1
HCC1937.rep2
HCC1954.rep2
HCC1954.rep1
MB231.rep2
MB231.rep1
MB436.rep2
MB436.rep1
76NF2V.rep2
76NF2V.rep1
MCF10A.rep2
MCF10A.rep1
Log2 Occupancy
4
3
2
1
0
Subtype
Normal
Luminal A
Luminal B
HER2
TNBC Claudin Low
TNBC Basal

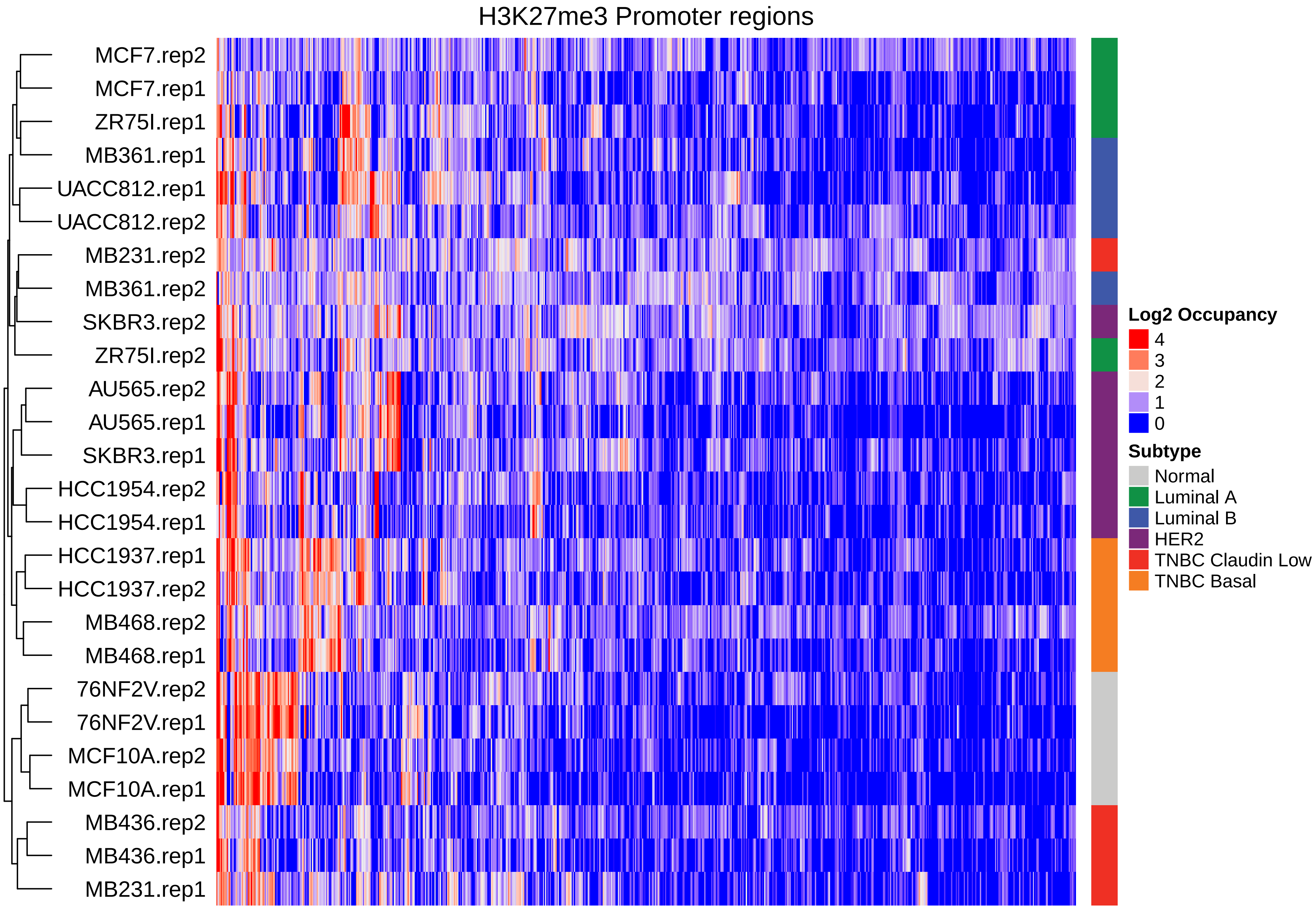

H3K27me3 Promoter regions
MCF7.rep2
MCF7.rep1
ZR75I.rep1
MB361.rep1
UACC812.rep1
UACC812.rep2
MB231.rep2
MB361.rep2
SKBR3.rep2
ZR75I.rep2
AU565.rep2
AU565.rep1
SKBR3.rep1
HCC1954.rep2
HCC1954.rep1
HCC1937.rep1
HCC1937.rep2
MB468.rep2
MB468.rep1
76NF2V.rep2
76NF2V.rep1
MCF10A.rep2
MCF10A.rep1
MB436.rep2
MB436.rep1
MB231.rep1
Log2 Occupancy
4
3
2
1
0
Subtype
Normal
Luminal A
Luminal B
HER2
TNBC Claudin Low
TNBC Basal

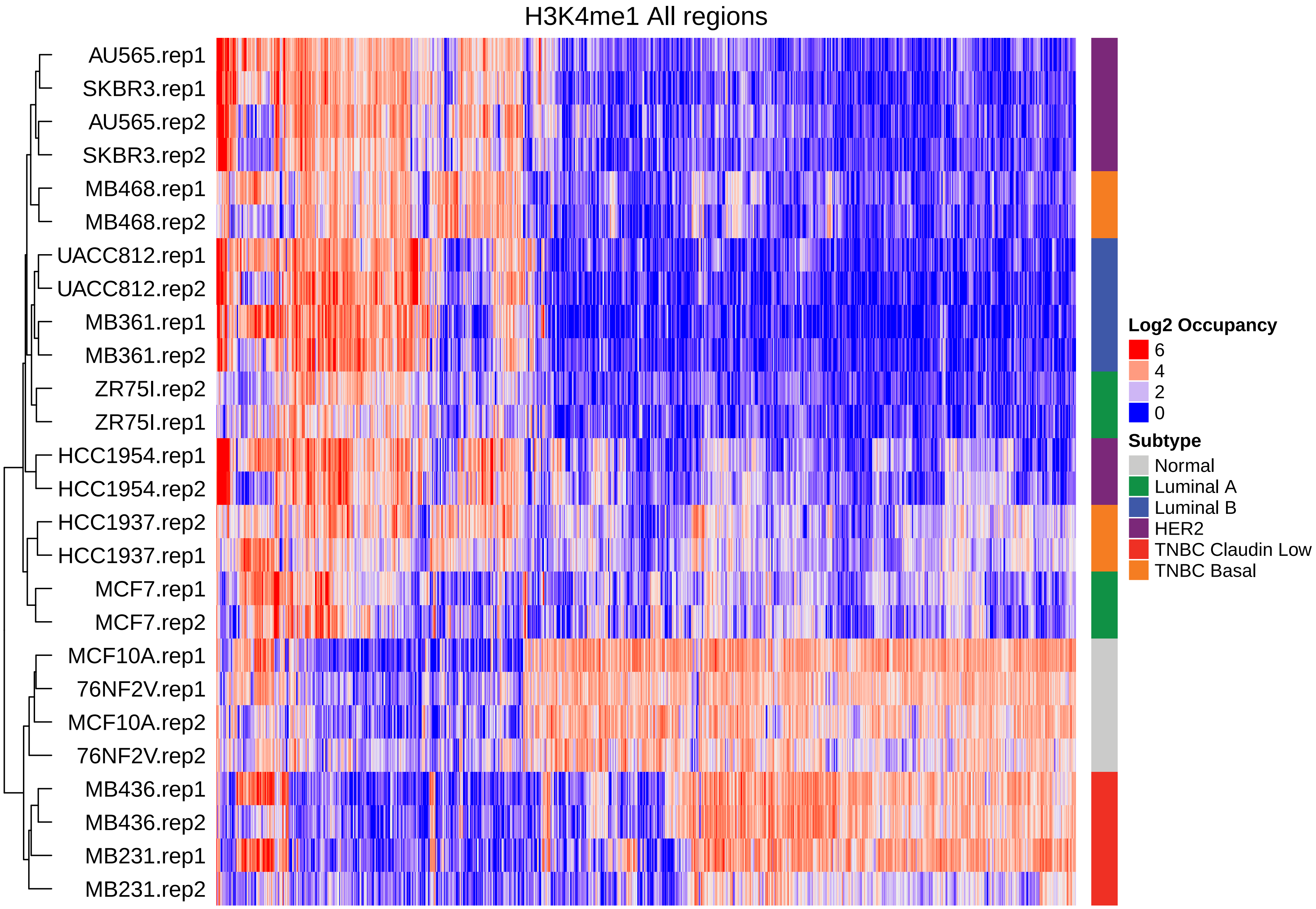
H3K4me1 All regions
AU565.rep1
SKBR3.rep1
AU565.rep2
SKBR3.rep2
MB468.rep1
MB468.rep2
UACC812.rep1
UACC812.rep2
MB361.rep1
MB361.rep2
ZR75I.rep2
ZR75I.rep1
HCC1954.rep1
HCC1954.rep2
HCC1937.rep2
HCC1937.rep1
MCF7.rep1
MCF7.rep2
MCF10A.rep1
76NF2V.rep1
MCF10A.rep2
76NF2V.rep2
MB436.rep1
MB436.rep2
MB231.rep1
MB231.rep2
Log2 Occupancy
6
4
2
0
Subtype
Normal
Luminal A
Luminal B
HER2
TNBC Claudin Low
TNBC Basal

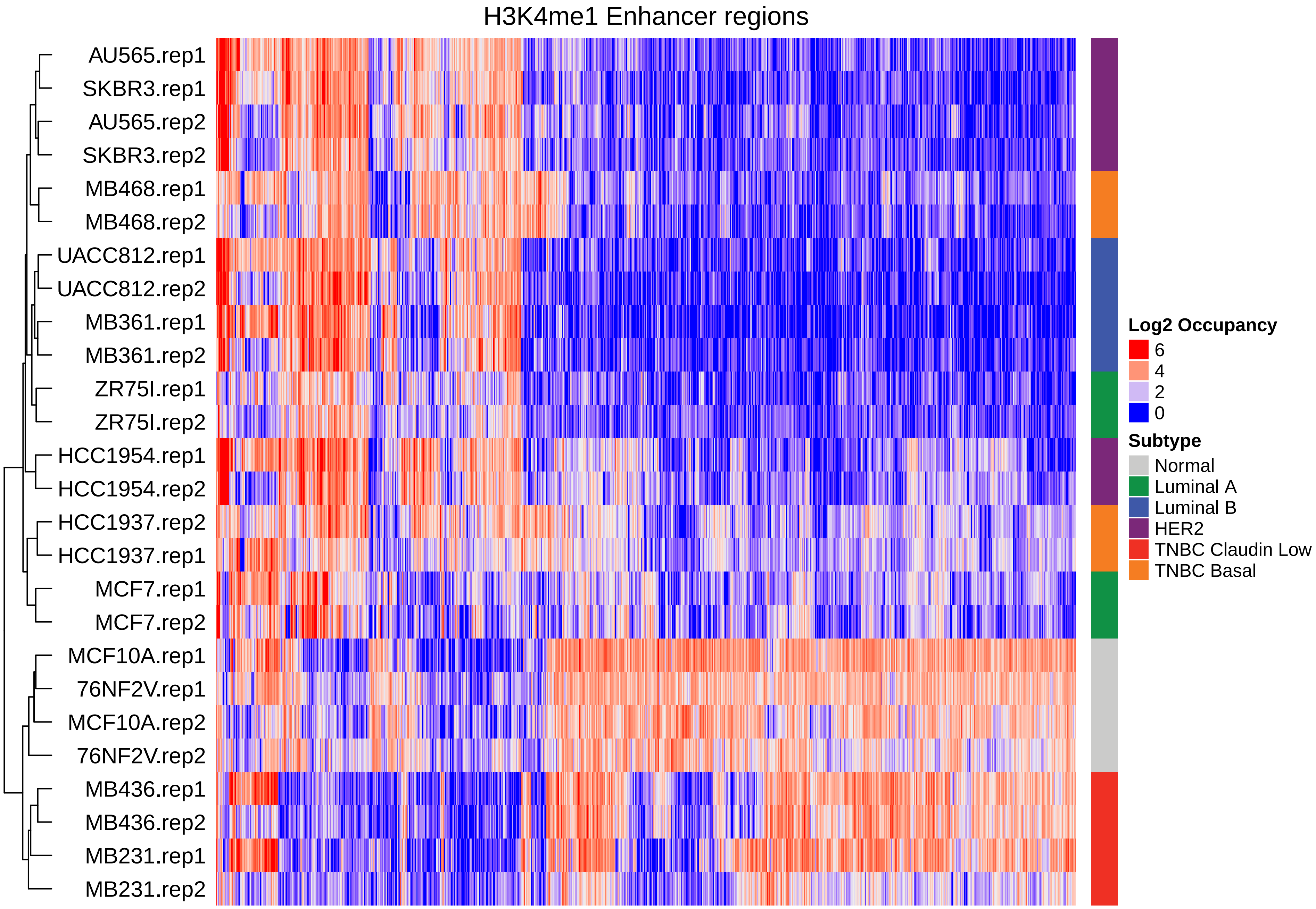
H3K4me1 Enhancer regions
AU565.rep1
SKBR3.rep1
AU565.rep2
SKBR3.rep2
MB468.rep1
MB468.rep2
UACC812.rep1
UACC812.rep2
MB361.rep1
MB361.rep2
ZR75I.rep1
ZR75I.rep2
HCC1954.rep1
HCC1954.rep2
HCC1937.rep2
HCC1937.rep1
MCF7.rep1
MCF7.rep2
MCF10A.rep1
76NF2V.rep1
MCF10A.rep2
76NF2V.rep2
MB436.rep1
MB436.rep2
MB231.rep1
MB231.rep2
Log2 Occupancy
6
4
2
0
Subtype
Normal
Luminal A
Luminal B
HER2
TNBC Claudin Low
TNBC Basal

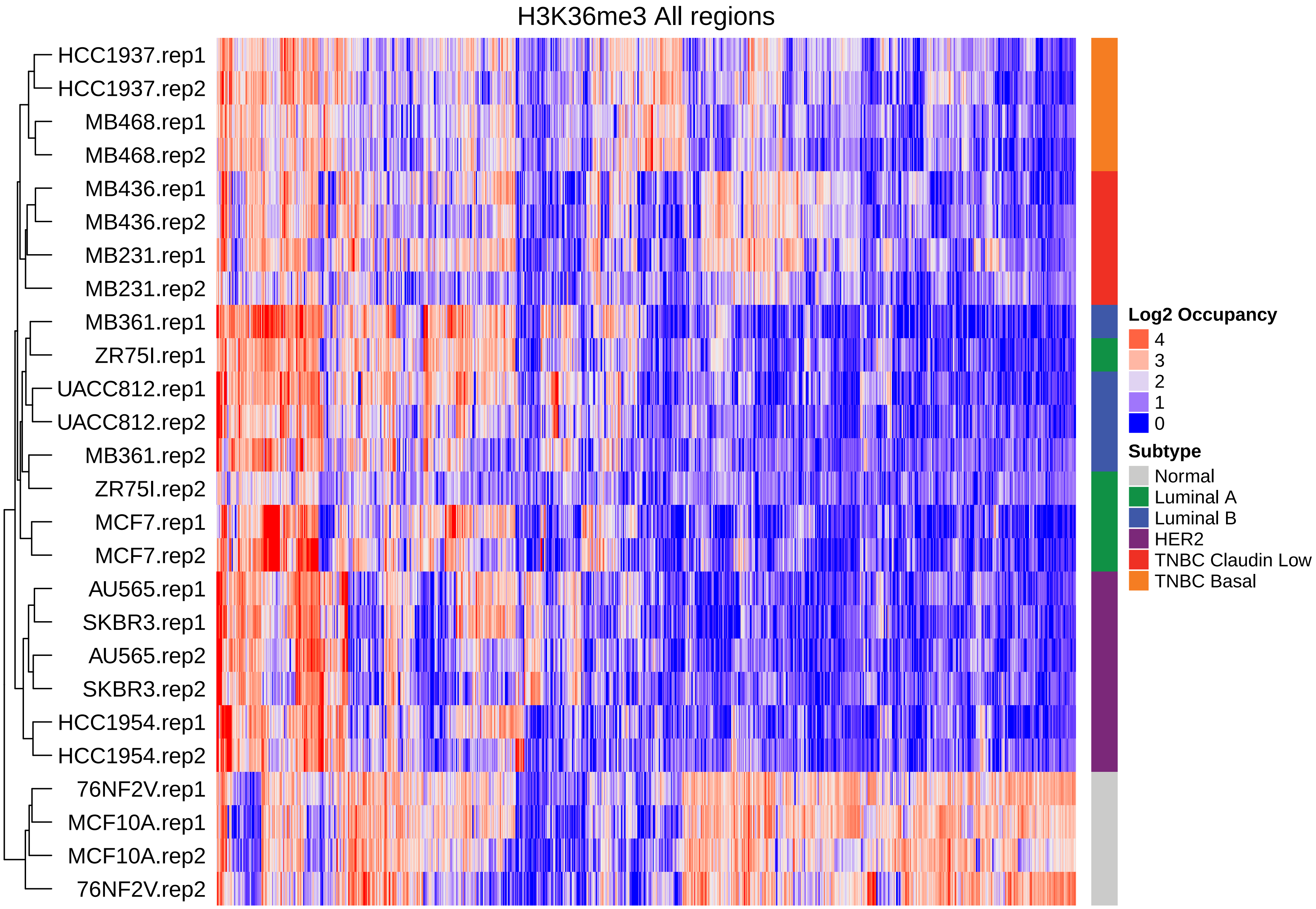
H3K36me3 All regions
HCC1937.rep1
HCC1937.rep2
MB468.rep1
MB468.rep2
MB436.rep1
MB436.rep2
MB231.rep1
MB231.rep2
MB361.rep1
ZR75I.rep1
UACC812.rep1
UACC812.rep2
MB361.rep2
ZR75I.rep2
MCF7.rep1
MCF7.rep2
AU565.rep1
SKBR3.rep1
AU565.rep2
SKBR3.rep2
HCC1954.rep1
HCC1954.rep2
76NF2V.rep1
MCF10A.rep1
MCF10A.rep2
76NF2V.rep2
Log2 Occupancy
4
3
2
1
0
Subtype
Normal
Luminal A
Luminal B
HER2
TNBC Claudin Low
TNBC Basal

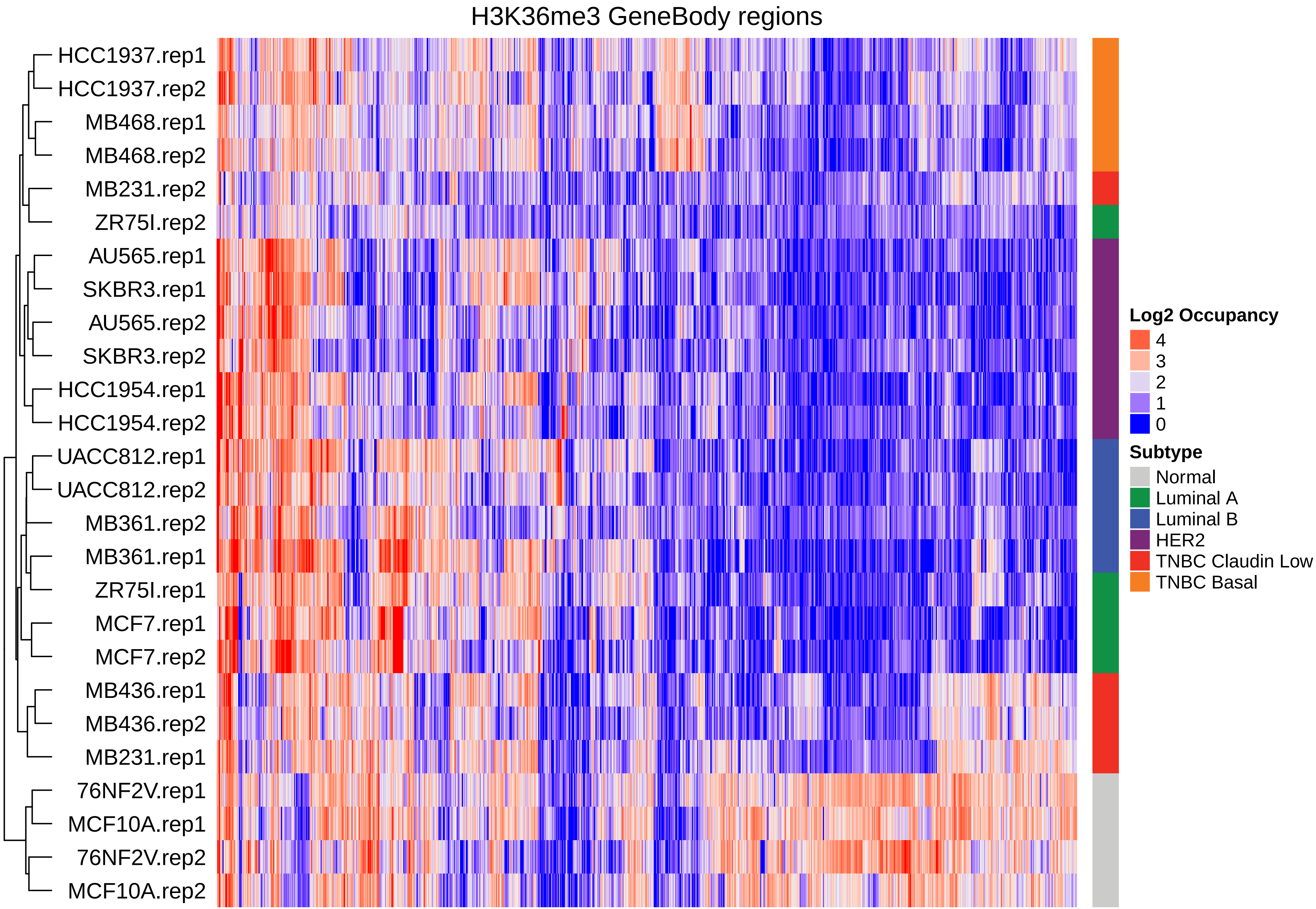

H3K36me3 GeneBody regions
HCC1937.rep1
HCC1937.rep2
MB468.rep1
MB468.rep2
MB231.rep2
ZR75I.rep2
AU565.rep1
SKBR3.rep1
AU565.rep2
SKBR3.rep2
HCC1954.rep1
HCC1954.rep2
UACC812.rep1
UACC812.rep2
MB361.rep2
MB361.rep1
ZR75I.rep1
MCF7.rep1
MCF7.rep2
MB436.rep1
MB436.rep2
MB231.rep1
76NF2V.rep1
MCF10A.rep1
76NF2V.rep2
MCF10A.rep2
Log2 Occupancy
4
3
2
1
0
Subtype
Normal
Luminal A
Luminal B
HER2
TNBC Claudin Low
TNBC Basal

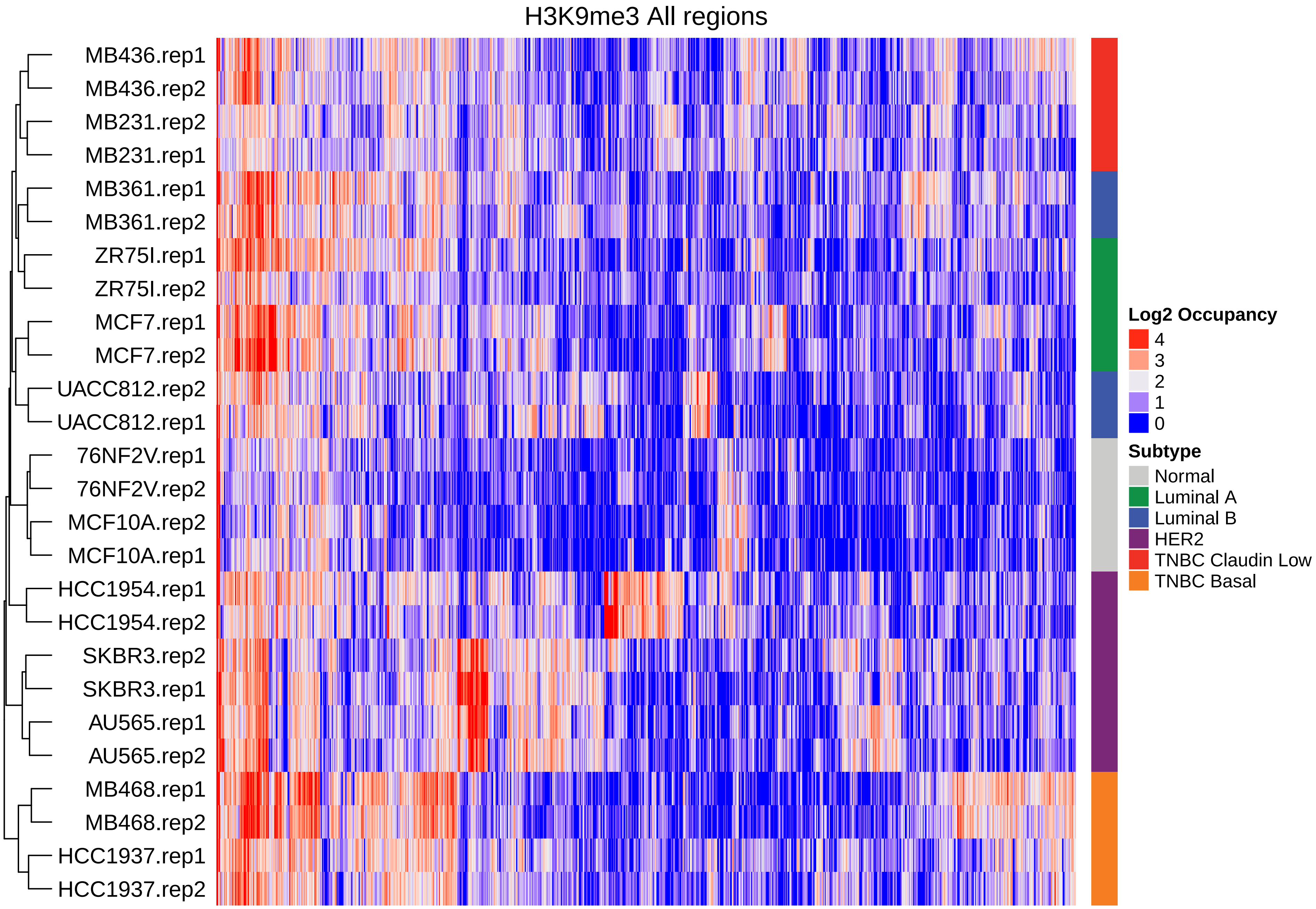
H3K9me3 All regions
MB436.rep1
MB436.rep2
MB231.rep2
MB231.rep1
MB361.rep1
MB361.rep2
ZR75I.rep1
ZR75I.rep2
MCF7.rep1
MCF7.rep2
UACC812.rep2
UACC812.rep1
76NF2V.rep1
76NF2V.rep2
MCF10A.rep2
MCF10A.rep1
HCC1954.rep1
HCC1954.rep2
SKBR3.rep2
SKBR3.rep1
AU565.rep1
AU565.rep2
MB468.rep1
MB468.rep2
HCC1937.rep1
HCC1937.rep2
Log2 Occupancy
4
3
2
1
0
Subtype
Normal
Luminal A
Luminal B
HER2
TNBC Claudin Low
TNBC Basal

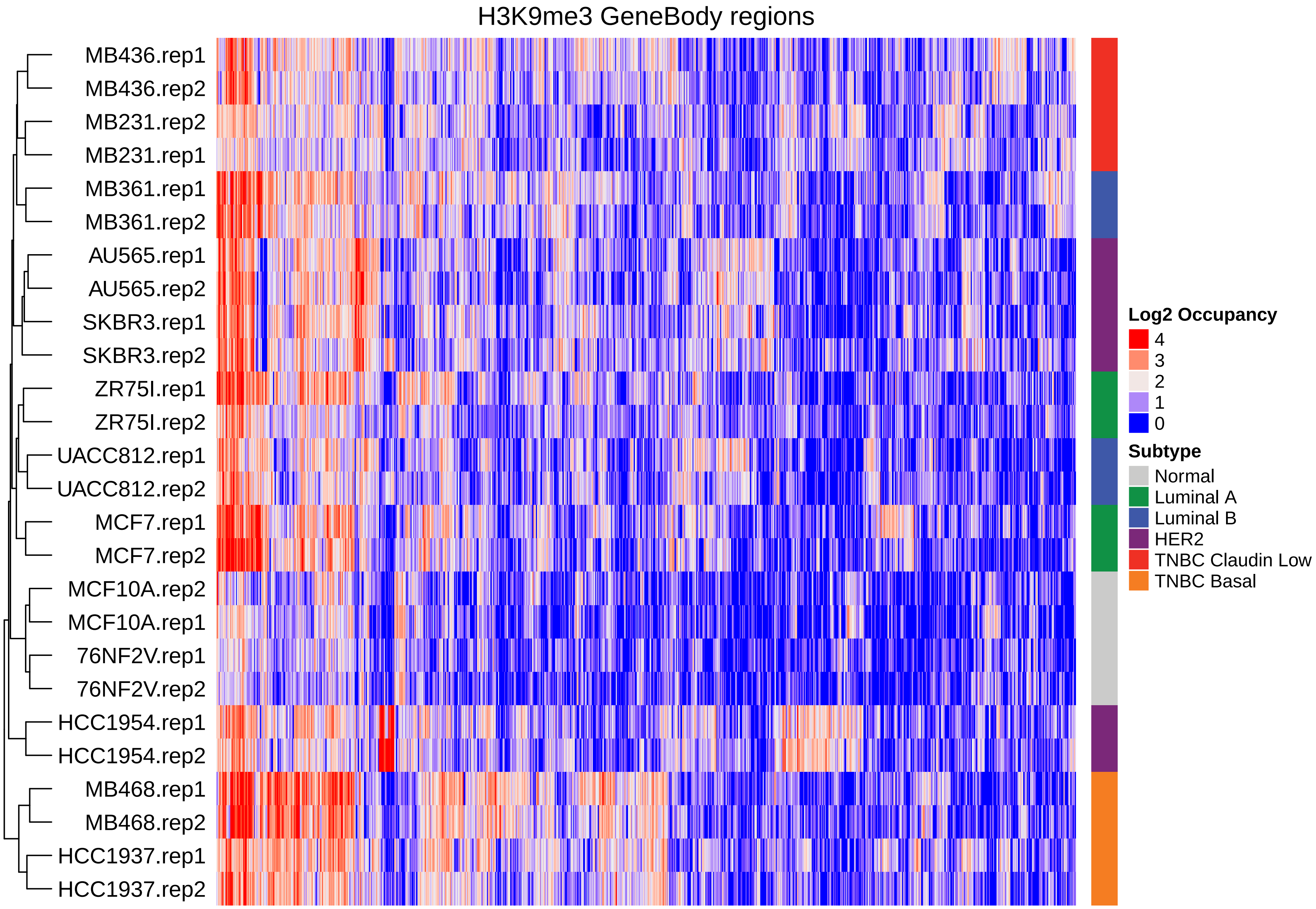
H3K9me3 GeneBody regions
MB436.rep1
MB436.rep2
MB231.rep2
MB231.rep1
MB361.rep1
MB361.rep2
AU565.rep1
AU565.rep2
SKBR3.rep1
SKBR3.rep2
ZR75I.rep1
ZR75I.rep2
UACC812.rep1
UACC812.rep2
MCF7.rep1
MCF7.rep2
MCF10A.rep2
MCF10A.rep1
76NF2V.rep1
76NF2V.rep2
HCC1954.rep1
HCC1954.rep2
MB468.rep1
MB468.rep2
HCC1937.rep1
HCC1937.rep2
Log2 Occupancy
4
3
2
1
0
Subtype
Normal
Luminal A
Luminal B
HER2
TNBC Claudin Low
TNBC Basal

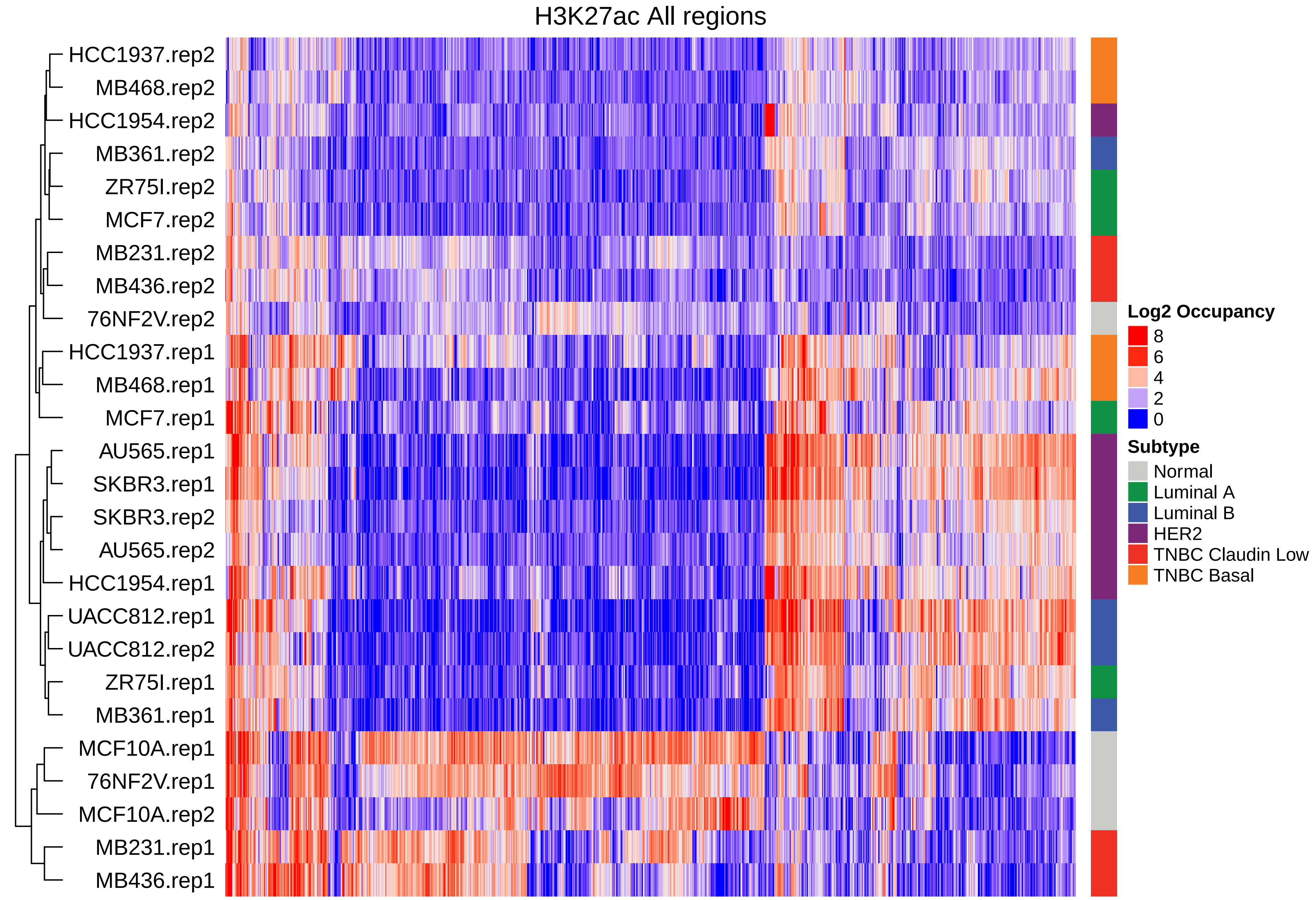
H3K27ac All regions
HCC1937.rep2
MB468.rep2
HCC1954.rep2
MB361.rep2
ZR75I.rep2
MCF7.rep2
MB231.rep2
MB436.rep2
76NF2V.rep2
HCC1937.rep1
MB468.rep1
MCF7.rep1
AU565.rep1
SKBR3.rep1
SKBR3.rep2
AU565.rep2
HCC1954.rep1
UACC812.rep1
UACC812.rep2
ZR75I.rep1
MB361.rep1
MCF10A.rep1
76NF2V.rep1
MCF10A.rep2
MB231.rep1
MB436.rep1
Log2 Occupancy
8
6
4
2
0
Subtype
Normal
Luminal A
Luminal B
HER2
TNBC Claudin Low
TNBC Basal

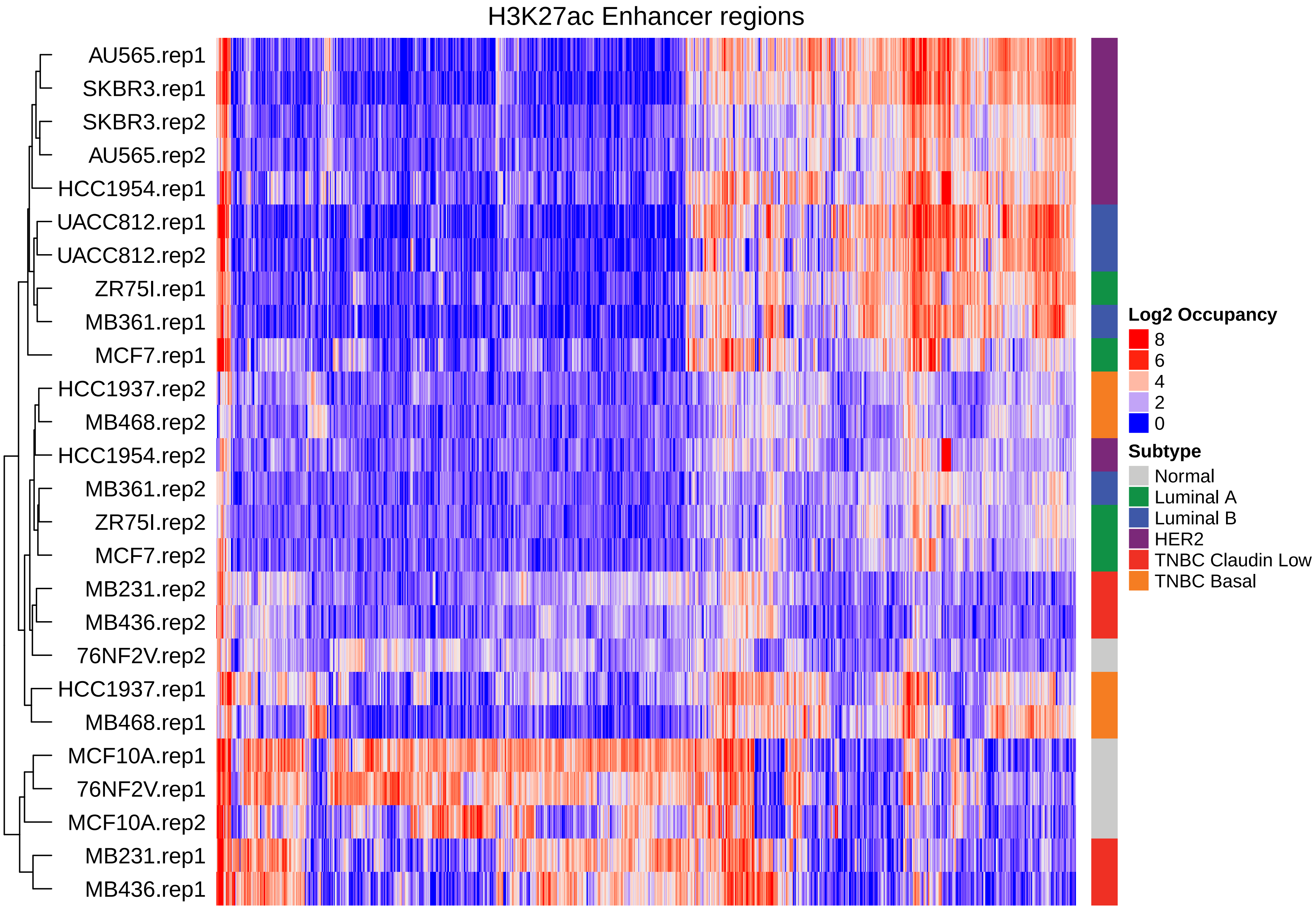
H3K27ac Enhancer regions
AU565.rep1
SKBR3.rep1
SKBR3.rep2
AU565.rep2
HCC1954.rep1
UACC812.rep1
UACC812.rep2
ZR75I.rep1
MB361.rep1
MCF7.rep1
HCC1937.rep2
MB468.rep2
HCC1954.rep2
MB361.rep2
ZR75I.rep2
MCF7.rep2
MB231.rep2
MB436.rep2
76NF2V.rep2
HCC1937.rep1
MB468.rep1
MCF10A.rep1
76NF2V.rep1
MCF10A.rep2
MB231.rep1
MB436.rep1
Log2 Occupancy
8
6
4
2
0
Subtype
Normal
Luminal A
Luminal B
HER2
TNBC Claudin Low
TNBC Basal

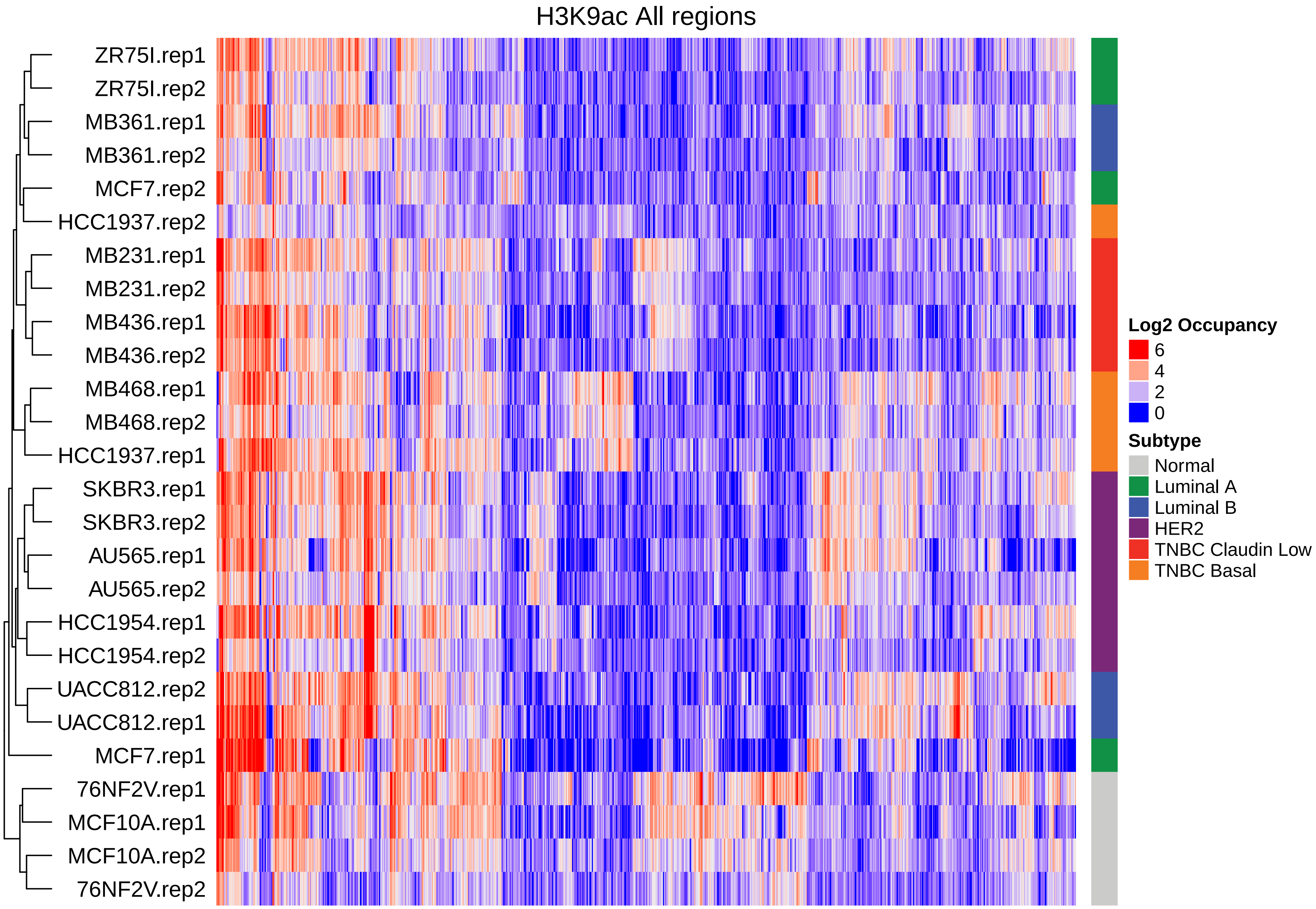

H3K9ac All regions
ZR75I.rep1
ZR75I.rep2
MB361.rep1
MB361.rep2
MCF7.rep2
HCC1937.rep2
MB231.rep1
MB231.rep2
MB436.rep1
MB436.rep2
MB468.rep1
MB468.rep2
HCC1937.rep1
SKBR3.rep1
SKBR3.rep2
AU565.rep1
AU565.rep2
HCC1954.rep1
HCC1954.rep2
UACC812.rep2
UACC812.rep1
MCF7.rep1
76NF2V.rep1
MCF10A.rep1
MCF10A.rep2
76NF2V.rep2
Log2 Occupancy
6
4
2
0
Subtype
Normal
Luminal A
Luminal B
HER2
TNBC Claudin Low
TNBC Basal

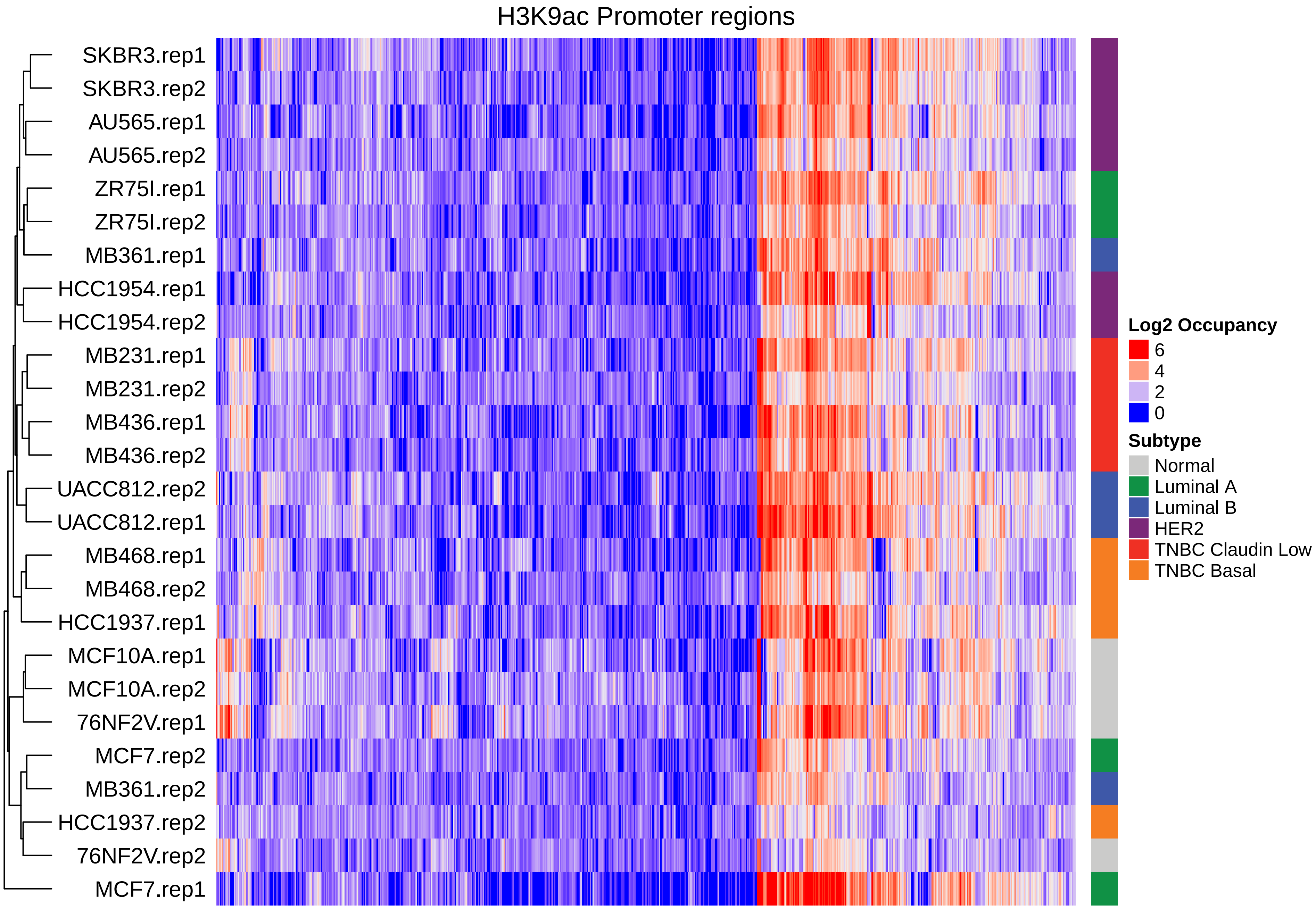
H3K9ac Promoter regions
SKBR3.rep1
SKBR3.rep2
AU565.rep1
AU565.rep2
ZR75I.rep1
ZR75I.rep2
MB361.rep1
HCC1954.rep1
HCC1954.rep2
MB231.rep1
MB231.rep2
MB436.rep1
MB436.rep2
UACC812.rep2
UACC812.rep1
MB468.rep1
MB468.rep2
HCC1937.rep1
MCF10A.rep1
MCF10A.rep2
76NF2V.rep1
MCF7.rep2
MB361.rep2
HCC1937.rep2
76NF2V.rep2
MCF7.rep1
Log2 Occupancy
6
4
2
0
Subtype
Normal
Luminal A
Luminal B
HER2
TNBC Claudin Low
TNBC Basal

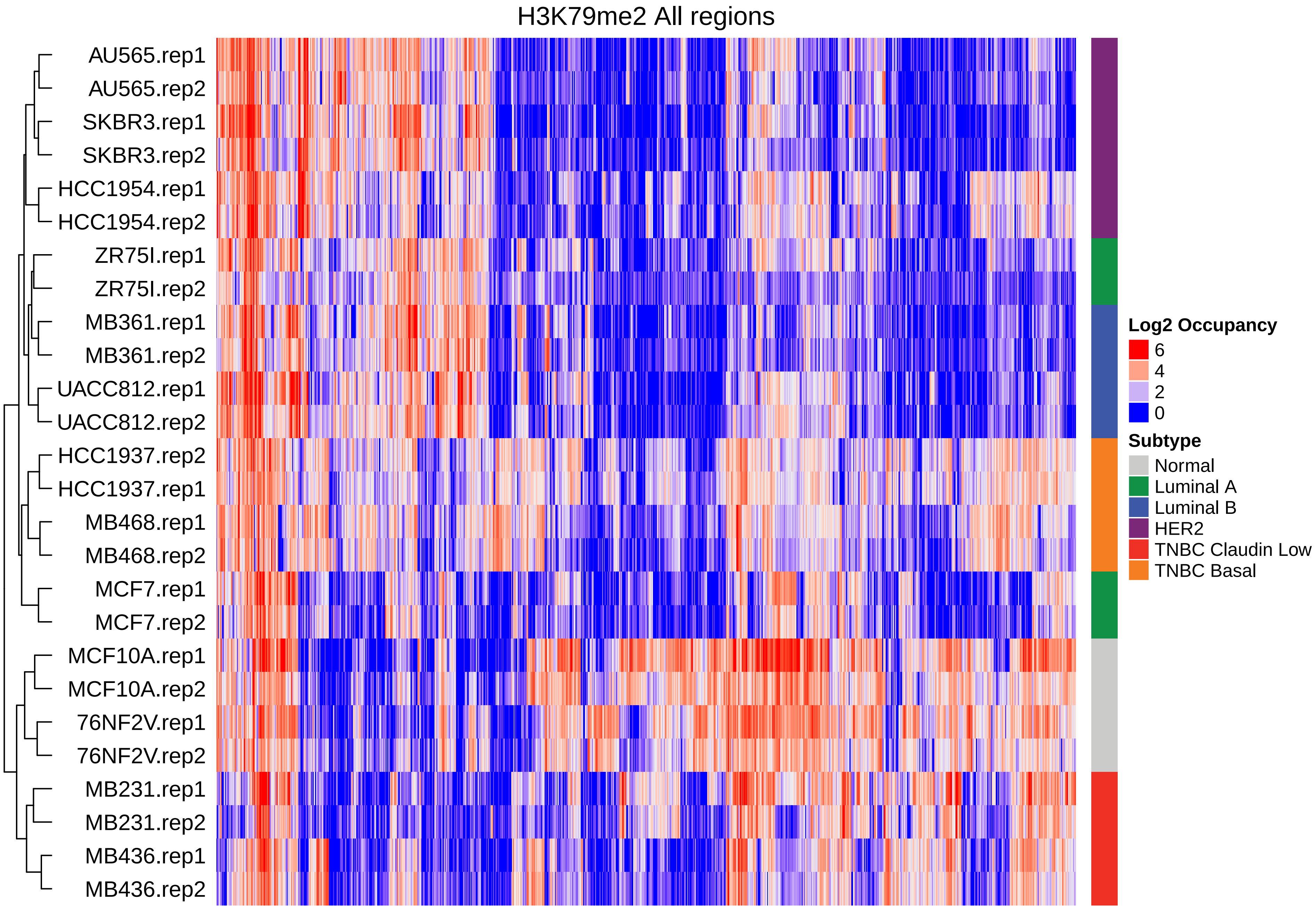

H3K79me2 All regions
AU565.rep1
AU565.rep2
SKBR3.rep1
SKBR3.rep2
HCC1954.rep1
HCC1954.rep2
ZR75I.rep1
ZR75I.rep2
MB361.rep1
MB361.rep2
UACC812.rep1
UACC812.rep2
HCC1937.rep2
HCC1937.rep1
MB468.rep1
MB468.rep2
MCF7.rep1
MCF7.rep2
MCF10A.rep1
MCF10A.rep2
76NF2V.rep1
76NF2V.rep2
MB231.rep1
MB231.rep2
MB436.rep1
MB436.rep2
Log2 Occupancy
6
4
2
0
Subtype
Normal
Luminal A
Luminal B
HER2
TNBC Claudin Low
TNBC Basal

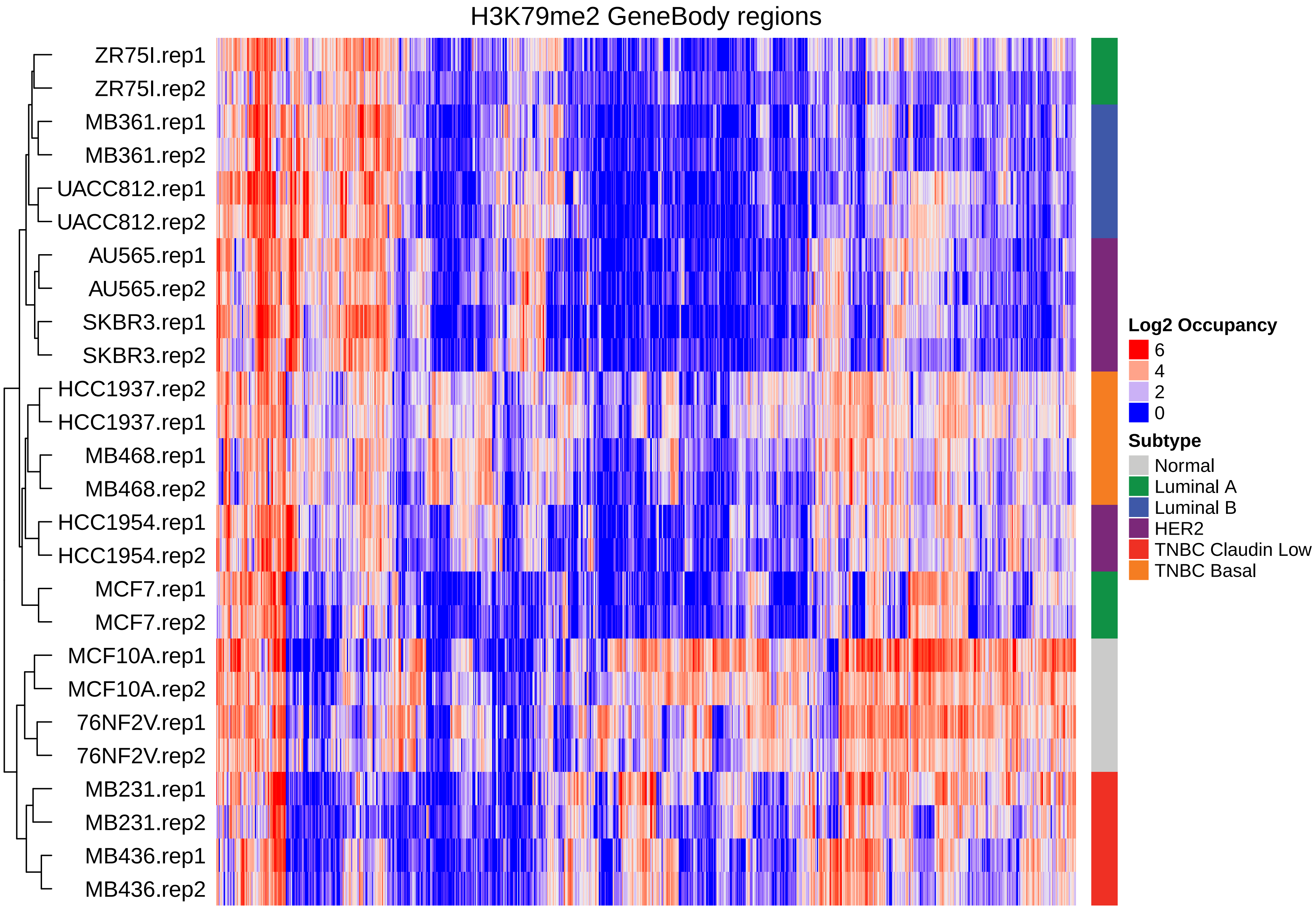
H3K79me2 GeneBody regions
ZR75I.rep1
ZR75I.rep2
MB361.rep1
MB361.rep2
UACC812.rep1
UACC812.rep2
AU565.rep1
AU565.rep2
SKBR3.rep1
SKBR3.rep2
HCC1937.rep2
HCC1937.rep1
MB468.rep1
MB468.rep2
HCC1954.rep1
HCC1954.rep2
MCF7.rep1
MCF7.rep2
MCF10A.rep1
MCF10A.rep2
76NF2V.rep1
76NF2V.rep2
MB231.rep1
MB231.rep2
MB436.rep1
MB436.rep2
Log2 Occupancy
6
4
2
0
Subtype
Normal
Luminal A
Luminal B
HER2
TNBC Claudin Low
TNBC Basal
